# Histone H3 Lysine 36 Trimethylation Is Established over the *Xist* Promoter by Antisense *Tsix* Transcription and Contributes to Repressing *Xist* Expression

**DOI:** 10.1128/MCB.00561-15

**Published:** 2015-10-16

**Authors:** Tatsuya Ohhata, Mika Matsumoto, Martin Leeb, Shinwa Shibata, Satoshi Sakai, Kyoko Kitagawa, Hiroyuki Niida, Masatoshi Kitagawa, Anton Wutz

**Affiliations:** a Department of Molecular Biology, Hamamatsu University School of Medicine, Hamamatsu, Japan; b WT and MRC Stem Cell Institute, University of Cambridge, Cambridge, United Kingdom; c Institute of Molecular Health Sciences, ETH, Zurich, Switzerland; d Department of Stem Cell Biology, Graduate School of Medicine, Kanazawa University, Kanazawa, Japan

## Abstract

One of the two X chromosomes in female mammals is inactivated by the noncoding *Xist* RNA. In mice, X chromosome inactivation (XCI) is regulated by the antisense RNA *Tsix*, which represses *Xist* on the active X chromosome. In the absence of *Tsix*, PRC2-mediated histone H3 lysine 27 trimethylation (H3K27me3) is established over the *Xist* promoter. Simultaneous disruption of *Tsix* and PRC2 leads to derepression of *Xist* and in turn silencing of the single X chromosome in male embryonic stem cells. Here, we identified histone H3 lysine 36 trimethylation (H3K36me3) as a modification that is recruited by *Tsix* cotranscriptionally and extends over the *Xist* promoter. Reduction of H3K36me3 by expression of a mutated histone H3.3 with a substitution of methionine for lysine at position 36 causes a significant derepression of *Xist*. Moreover, depletion of the H3K36 methylase *Setd2* leads to upregulation of *Xist*, suggesting H3K36me3 as a modification that contributes to the mechanism of *Tsix* function in regulating XCI. Furthermore, we found that reduction of H3K36me3 does not facilitate an increase in H3K27me3 over the *Xist* promoter, indicating that additional mechanisms exist by which *Tsix* blocks PRC2 recruitment to the *Xist* promoter.

## INTRODUCTION

In mammals, X chromosome inactivation (XCI) provides dosage compensation between the sexes for X-linked genes (1). The noncoding RNA (ncRNA) *Xist* initiates chromosome-wide inactivation of one of the two X chromosomes of female cells. On the active X chromosome in males and females, *Xist* is repressed by several mechanisms. In mice, the *Tsix* ncRNA is transcribed over the *Xist* locus in the antisense orientation and functions as a repressor of *Xist* on the chromosome from which it is transcribed (2). The function of *Tsix* has been extensively studied in mouse embryonic stem (ES) cells, which constitute a model for studying the initiation of random XCI (1, 3–5). Disruption of *Tsix* leads to derepression of *Xist* whose extent varies with experimental details in a number of different studies (6–9). In mouse preimplantation development, imprinted XCI leads to inactivation of the paternally inherited X chromosome in female embryos. Overexpression of *Tsix* from the paternal X chromosome prevents XCI and causes lethality (10). Conversely, disruption of *Tsix* on the maternally inherited X chromosome in males and females causes lethality due to misregulation of imprinted XCI in the extraembryonic lineages (11, 12). However, in the embryonic lineages, the *Tsix* disruption-bearing X chromosome is fated to become the inactive X chromosome (Xi) (6, 12).

Mutation of *Tsix* causes death of male embryos due to initiation of X inactivation in extraembryonic tissues. This lethality can be prevented by complementing the extraembryonic lineages, suggesting that *Tsix*-independent mechanisms can act to repress *Xist* in the embryonic lineages (13). *Tsix*-independent mechanisms can also be inferred from other mammals, including humans, which lack a functionally conserved *Tsix* homologue (14). Our previous work linked *Tsix*-independent *Xist* repression to Polycomb repressive complex 2 (PRC2) (15). PRC2 contains the Polycomb genes *Eed* and *Suz12* and the SET domain histone H3 methyltransferase gene *Ezh2. Eed* is required for PRC2-mediated trimethylation of histone H3 lysine 27 (H3K27me3) (16). Combined mutations in *Tsix* and *Eed* lead to deregulation of *Xist* in male ES cells, leading to activation of *Xist* in a majority of the cells (15). Although it appears that *Tsix* and PRC2 act in parallel to repress *Xist*, the precise function of Polycomb complexes in repressing *Xist* remains to be established. Notably, transient enrichment of H3K27me3 on the *Xist* promoter has also been proposed as one of the sequential events for *Xist* activation (17). However, PRC2 is generally correlated with repression of genes, and no molecular mechanism for an activating function has been identified yet. Additional indirect effects of PRC2 disruption also cannot be ruled out.

Several regulators of *Xist* have been identified, including the X-linked *Rnf12*, *Ftx*, and *Jpx* genes. Rnf12 inhibits *Xist* repression in part through targeting Rex1 protein for degradation (4, 18). Several transcription factors associated with ES cell pluripotency, including Oct4, Sox2, Nanog, and Rex1, have been proposed to be implicated in the repression of *Xist* in ES cells (3, 19, 20), but their precise function in the embryos remains to be resolved (21, 22). Recently, the activation of *Xist* during the progression from naive to primed pluripotency of mouse ES cells was examined in detail in chemically defined medium (5). *Ftx* and *Jpx* are ncRNA genes which are located upstream of *Xist* and positively regulate *Xist. Jpx* may function through evicting Ctcf and changing chromatin conformation (23, 24). Mutation of *Ftx* leads to decreased *Xist* expression in ES cells (25), but *Ftx* is dispensable for imprinted XCI in embryos (26). Furthermore, a number of studies have suggested that changes in chromatin organization and pairing of the X chromosomes along the X chromosome inactivation center (*Xic*) regions contributes to the regulation of XCI (27–29). Taken together, these studies illustrate that multiple factors interact in the regulation of *Xist*.

Here, we investigated repressive mechanisms of *Xist* in male ES cells, which possess a single X chromosome, and thus, *trans* interactions and pairing are not expected to be relevant. We show that genetic disruption of *Eed* and *Tsix* leads to loss of *Xist* repression despite the presence of other regulators of *Xist*, including Rnf12, Nanog, and Oct4. Moreover, DNA methylation and PRC2 recruitment are not essential for *Xist* repression as long as *Tsix* transcription is unperturbed. We show that *Tsix* transcription induces trimethylation of histone H3 lysine 36 (H3K36me3) at the *Xist* promoter, which contributes to the repression of *Xist* expression, among other mechanisms.

## MATERIALS AND METHODS

### Cell culture and generation of ES cell lines.

Details of plasmid construction and *Setd2* knockdown are provided in the supplemental material. ES cells were cultured as previously described (30, 31). The *Dnmt* triple-knockout (Dnmt TKO) ES cells were a gift from Masaki Okano (Kumamoto University, Japan) (32). For generating R^−/−^ Δ*Tsix* cells, the conditions for electroporation, antibiotic selection, Southern hybridization, and removing the selection cassette by transient expression of Cre recombinase were described previously (33). The expression vector pPyCAG-EGFP-IZ (34) was a gift from Hitoshi Niwa (Kumamoto University, Japan) and provided by the RIKEN BRC through the National Bio-Resource Project of the MEXT, Japan. For generating enhanced green fluorescent protein (EGFP), H3.3wt, and K36M transgenic lines, 10 μg of each expression vectors pPyCAG-EGFP-IZ, pPyCAG-H3.3wt-FH-IZ, and pPyCAG-K36M-FH-IZ was linearized by SalI, purified, and transfected into 2 million J1 ES cells with a Neon electroporator (Invitrogen, CA). The settings were as follows: 1,400 V; pulse width, 10 ms; 3 times. The cells were selected with 300 μg/ml zeocin, and a pool of approximately 100 clones of transgene-expressing cells was used for the experiments.

### ChIP.

Three different chromatin immunoprecipitation (ChIP) methods were used: the H2AK119ub1-formulated method (see Fig. 4E) and the standard method without nuclear isolation (see Fig. 4D and 6C) and with nuclear isolation especially for improving the immunoprecipitation efficiency for nuclear transcription factors (see Fig. 2B and C, 3B and C, 5B, 6B, and 7C and D). ChIP for H2AK119ub1 was performed as previously described (35) with some modifications. The details of the procedures are provided in the supplemental materials and methods. The following antibodies were used: anti-H2AK119ub1 (05-678; Upstate Biotechnology, NY) and normal mouse IgM (M5909; Sigma-Aldrich, MO) as a mock-immunoprecipitated control (refered to as “mock”). A standard ChIP method without nuclear isolation was performed as previously described (36) except that a Covaris S1 system (Covaris Inc., MA) was used for DNA fragmentation, using the following settings: 20% duty, intensity of 10.0, 500 cycles/burst, 360-s duration, and 1 cycle. The following antibodies were used: anti-H3K27me3 (07-449; Upstate), anti-H3K36me3, (ab9050; Abcam, Cambridge, United Kingdom), and normal rabbit IgG (I5006; Sigma-Aldrich) as a mock control. The ChIP method with nuclear isolation was performed as previously described (37). The following antibodies were used: anti-Nanog (8822; CST, MA), anti-Oct4 (5677; CST), anti-H3K4me2 (CMA303; a gift from Hiroshi Kimura) (38), anti-H3K27me3 (CMA323; a gift from Hiroshi Kimura) (38), anti-H3K36me3 (ab9050; Abcam), and normal rabbit IgG (I5006; Sigma-Aldrich) as a mock control.

### Quantitative real-time PCR.

Total RNA was purified with RNeasy minikit (Qiagen, Hilden, Germany) and cDNA was generated by reverse transcriptase SuperScript II (Invitrogen, MA). Quantitative real-time PCR for gene expression and ChIP was performed using iQ SYBR green Supermix (Bio-Rad, CA) with a single-color detection MyIQ i cycler (Bio-Rad), SYBR green PCR master mix (Life Technologies, California, USA), or Thunderbird SYBR qPCR mix (Toyoko, Osaka, Japan) with a StepOnePlus real-time PCR system (Life Technologies). Primer sequences covering the entire *Xist*/*Tsix* transcription unit (see Fig. 6A) were described previously (39), and those used for quantitative real-time PCR for both RNA expression and ChIP analyses are listed in Table S1 in the supplemental material.

### RNA-FISH.

RNA fluorescence *in situ* hybridization (RNA-FISH) was performed according to the protocols described previously (10). The strand-specific RNA-FISH probe was generated as previously described (40).

### Immunostaining and Western blotting.

Methods for immunostaining and Western blotting were performed as described previously (33). The antibody for immunostaining was Ring1b (gift from Haruhiko Koseki) (41). The antibodies for Western blotting were H3K36me3 (ab9050; Abcam), Flag (A8692; Sigma-Aldrich), H2AK119ub1 (05-678; Upstate), H3 (see Fig. S1G in the supplemental material) (ab1791; Abcam), H3 (see Fig. 4D) (39763; Active Motif), H3K27me3 (see Fig. S1G in the supplemental material) (07-449; Upstate), H3K27me3 (see Fig. 4D) (gift from Hiroshi Kimura; CMA323) (38).

### Bisulfite sequencing.

DNA methylation analysis by bisulfite sequencing was performed following the manufacturer's protocol (Imprint DNA modification kit MOD50; Sigma-Aldrich) as described previously (10).

## RESULTS

### Eed is required for *Tsix*-independent *Xist* repression in naive ES cells.

**FIG 1:**
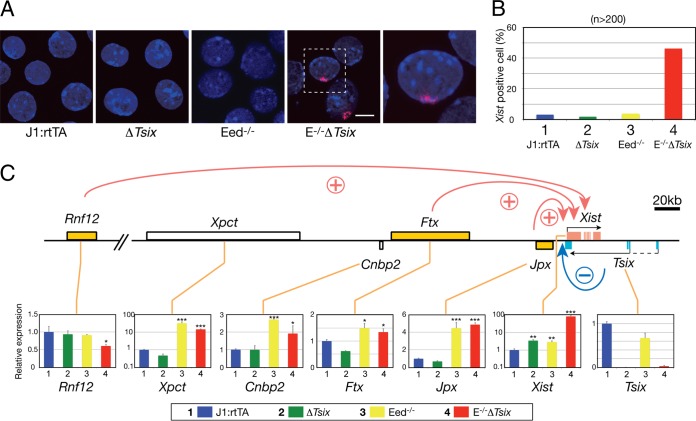
H3K27me3 is required for *Tsix*-independent *Xist* repression in naive ES cells. (A) *Xist* RNA-FISH (red) in J1:rtTA control, Δ*Tsix*, Eed^−/−^, and E^−/−^ Δ*Tsix* ES cells (the boxed area is magnified in the rightmost panel). Nuclei were counterstained with DAPI (blue). Bar, 10 μm. (B) Percent *Xist*-positive cells (*n* > 200). (C) Map of the X chromosome inactivation center (*Xic*) and its linked genes *Rnf12*, *Xpct*, *Cnbp2*, *Ftx*, *Jpx*, *Xist*, and *Tsix. Rnf12*, *Ftx*, and *Jpx* are reported as *Xist* activators. Their expression was measured by qRT-PCR. Expression is shown relative to undifferentiated J1:rtTA ES cells and normalized to *Gapdh* (*n* = 3). All cells were cultured with 2i medium. *, *P* < 0.05; **, *P* < 0.01; ***, *P* < 0.001 (Student's *t* test).

**FIG 2:**
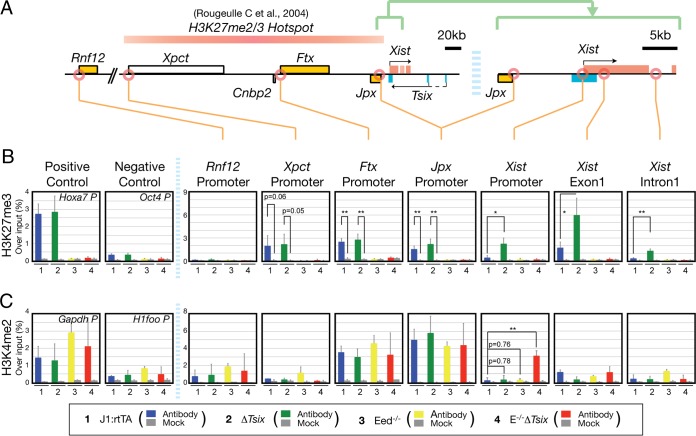
*Tsix* prevents H3K27me3 invasion from the hot spot in naive ES cells. (A) Map of *Xic*. The H3K27me2/3 hot spot (43), in the 340-kb region 5′ of *Xist*, is shown. (B and C) ChIP analysis of H3K27me3 (B) and H3K4me2 (C) across the *Xic* locus. *Hoxa7* and *Oct4* promoters for H3K27me3 and *Gapdh* and *H1foo* promoters for H3K4me2 were used as positive- and negative-control loci, respectively. Data are means and standard deviations from three independent experiments. All cells were cultured with 2i medium. *, *P* < 0.05; **, *P* < 0.01 (Student's *t* test).

Recently, the use of chemically defined culture conditions for investigating XCI has been explored (5). Naive pluripotent mouse ES cells (31) cultured in the presence of MAP kinase inhibitors and GSK3 kinase inhibitors (2i) have reduced promoter-specific Polycomb complex-associated histone modifications (37) and DNA methylation, which is likely to resemble the actual situation in the developing epiblast (42). We reasoned that under 2i culture conditions, the effects of chromatin on *Xist* repression could be discerned from indirect effects on the pluripotent state more readily. We analyzed *Xist* expression in naive male ES cells that harbor disruptions of *Eed* (Eed^−/−^) and *Tsix* (Δ*Tsix*) (see Fig. S1E in the supplemental material) (15) by RNA-FISH (Fig. 1A and B) and qRT-PCR (Fig. 1C). J1:rtTA ES cells were used as a parental control cell line (15). A significant derepression of *Xist* was observed in Δ*Tsix* and Eed^−/−^ cells by semiquantitative PCR (Fig. 1C, *Xist* [Δ*Tsix*, 3.47 ± 0.46; Eed^−/−^, 2.93 ± 0.39; *P* < 0.01]). However, the number of *Xist* clusters observed with *Xist* RNA-FISH was not significantly increased in Δ*Tsix* and Eed^−/−^ cells over the control cell line J1:rtTA (Fig. 1B [J1:rtTA, 3.2%; Δ*Tsix*, 1.9%; Eed^−/−^, 3.9%]). In contrast, combined disruption of both *Eed* and *Tsix* resulted in the appearance of *Xist* clusters in 46.2% of E^−/−^ Δ*Tsix* ES cells (Fig. 1B) and high levels of *Xist* expression (Fig. 1C, *Xist* [E^−/−^ Δ*Tsix*, 81.73 ± 15.33; *P* < 0.001]). Furthermore, the active histone mark H3K4me2 was strongly increased over the *Xist* promoter in Eed^−/−^ Δ*Tsix* cells (Fig. 2C [E^−/−^ Δ*Tsix*, 13.16 ± 0.51; *P* < 0.01]), in contrast to cells with mutation of either *Tsix* or *Eed* (Fig. 2C [Δ*Tsix*, 1.40 ± 0.40; Eed^−/−^, 1.26 ± 0.33; *P* = 0.78 and *P* = 0.76, respectively]). We conclude that PRC2 function is required for *Tsix*-independent *Xist* repression in naive pluripotent cells, a finding that is consistent with earlier observations in serum-LIF-cultured ES cells (15). We did not observe an increase of H3K4me2 on the *Xist* promoter, when *Tsix* was truncated in either in naive ES cells (Fig. 2C) or ES cells that were cultured in serum-LIF-based medium (15), contrasting a previous report on serum- and LIF-cultured ES cells (39). We attribute this apparent difference to the use of different ES cell lines and different strategies for mutating *Tsix*, where we have chosen an insertion of a gene trap cassette that truncates *Tsix* transcripts before the *Xist* gene locus.

### The Xist regulators *Ftx* and *Jpx* but not *Rnf12* are elevated in *Eed*-deficient ES cells.

To investigate the mechanisms of *Xist* activation in Eed^−/−^ Δ*Tsix* cells further, we analyzed the expression of *Rnf12*, *Ftx*, and *Jpx* and two additional genes within the X chromosome inactivation center (*Xic*), *Xpct* and *Cnbp2*. Increased *Rnf12* expression is correlated with activation of *Xist* (18, 21) but was decreased in Eed^−/−^ Δ*Tsix* cells (Fig. 1C, *Rnf12* [E^−/−^ Δ*Tsix*, 0.60 ± 0.05; *P* < 0.05]). This observation excludes upregulation of Rnf12 as a cause of *Xist* activation in E^−/−^ Δ*Tsix* cells. The observed repression of *Rnf12* could likely be a consequence of the activation of *Xist* in Eed^−/−^ Δ*Tsix* cells. *Ftx* and *Jpx* are ncRNAs that are located upstream of *Xist* and have also been proposed to positively regulate *Xist* RNA expression (23–25). We observed increased *Ftx* and *Jpx* expression in *Eed*-deficient ES cells (Fig. 1C, *Ftx* [Eed^−/−^, 1.50 ± 0.18; E^−/−^ Δ*Tsix*, 1.34 ± 0.12; *P* < 0.05] and *Jpx* [Eed^−/−^, 4.47 ± 0.63; Eed^−/−^ Δ*Tsix*, 4.86 ± 0.17; *P* < 0.001]). Similarly, *Xpct* and *Cnbp2* expression was also increased, showing that lack of *Eed* caused derepression of multiple genes within the *Xic* region (Fig. 1C, *Xpct* [Eed^−/−^, 31.59 ± 4.75; E^−/−^ Δ*Tsix*, 14.24 ± 0.97; *P* < 0.001] and *Cnbp2* [Eed^−/−^, 2.70 ± 0.04 {*P* < 0.001}; E^−/−^ Δ*Tsix*, 1.91 ± 0.44 {*P* < 0.05}).

We next investigated the distribution of H3K27me3 across the *Xic* region (Fig. 2A). In J1:rtTA and Δ*Tsix* cells, H3K27me3 could be detected at the *Xpct*, *Ftx*, and *Jpx* promoters, which are located within a 340-kb region 5′ of *Xist* that has been previously characterized as a hot spot region harboring high levels of H3K27me3 and H3K27me2 (Fig. 2B, *Xpct*, *Ftx*, and *Jpx* promoters) (43). An H3K27me3 hot spot was prominent in ES cells cultured in 2i and serum-based medium and encompassed the *Xpct*, *Ftx*, and *Jpx* promoters. As expected, H3K27me3 was lost in *Eed*^−/−^ and E^−/−^ Δ*Tsix* cells (Fig. 2B, *Xpct*, *Ftx*, and *Jpx* promoters). This finding suggests that the expression of *Xic*-linked genes *Xpct*, *Cnbp2*, *Ftx*, and *Jpx* is repressed (Fig. 1C) by PRC2 and H3K27me3 spreading from the hot spot (Fig. 2B). Interestingly, the expression of the hot spot-linked genes *Xpct* and *Cnbp2* was decreased in Eed^−/−^ Δ*Tsix* cells compared with *Eed*^−/−^ cells (Fig. 1C), suggesting that these genes are potentially also partially repressed by ectopically expressed *Xist* in cells lacking *Tsix* and *Eed*. However, this repressive effect of *Xist* activation is small compared with the derepression observed after loss of *Eed*, such that a net activation is observed between wild-type control and Eed^−/−^ Δ*Tsix* cells.

We next investigated whether *Tsix* prevented H3K27me3 spreading over the *Xist* promoter from the hot spot in J1:rtTA-naive ES cells. H3K27me3 was enriched at the *Xpct*, *Ftx*, and *Jpx* promoters (Fig. 2B) (*Xpct*, 1.99% ± 1.30% of input; *Ftx*, 2.49% ± 0.45% of input; *Jpx*, 1.51% ± 0.37% of input) but not at the *Xist* promoter (Fig. 2B) (0.41% ± 0.32% of input), suggesting a boundary of the H3K27me3 hot spot between the *Jpx* and *Xist* promoters. In Δ*Tsix* ES cells, H3K27me3 extended over the *Xist* promoter and gene body (Fig. 2B [*Xist* promoter, 5.44- ± 1.77-fold change {*P* < 0.05}; *Xist* exon 1, 3.49- ± 1.29-fold change {*P* < 0.05}; *Xist* intron 1, 4.14- ± 0.81-fold change {*P* < 0.01}] [enrichment is measured relative to J1:rtTA]). This observation showed that *Tsix* prevented the spreading of H3K27me3 from the hot spot into the *Xist* locus, which is consistent with earlier results in serum and LIF cultures (44).

### Nanog and Oct4 binding does not prevent *Xist* activation in *Eed*- and *Tsix*-deficient cells.

**FIG 3:**
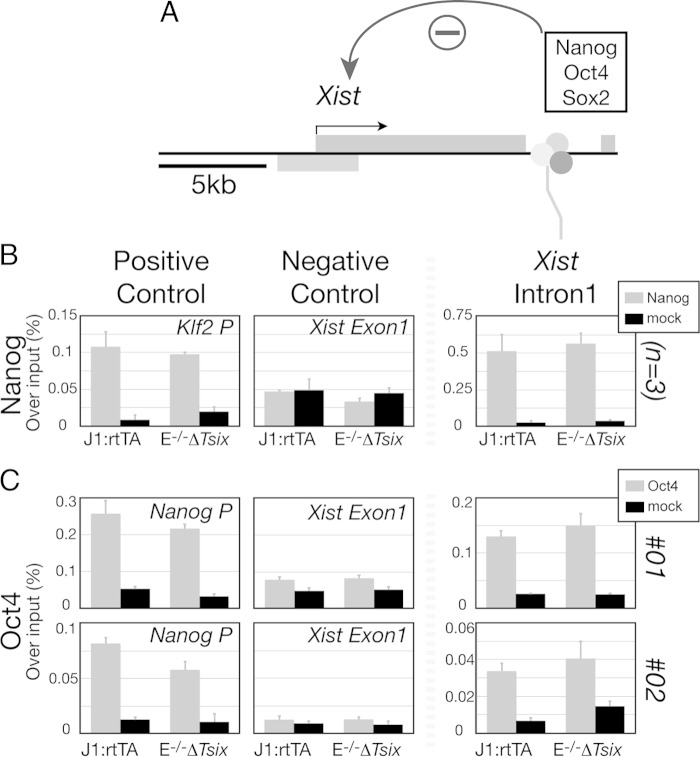
Nanog and Oct4 enrichment is not changed in Eed^−/−^ Δ*Tsix* cells. (A) Transcription factors associated with pluripotency, including Nanog, Oct4, and Sox2, are proposed as *Xist* repressors through a binding site within *Xist* intron 1. (B and C) Chromatin recruitment of the putative *Xist* repressors Nanog (B) and Oct4 (C) at its binding site at *Xist* intron 1, measured by ChIP. *Klf2* promoter and *Xist* exon 1 for Nanog and *Nanog* promoter and *Xist* exon 1 for Oct4 were used as positive- and negative-control loci, respectively. For Nanog, data are means and standard deviations from three independent experiments. For Oct4, data from two independent experiments are shown. All cells were cultured with 2i medium.

A number of recent studies have implicated the binding of transcription factors, including Nanog and Oct4, in *Xist* repression through a prominent binding site within *Xist* intron 1 (Fig. 3A) (19). To assess whether these pluripotency factors remain bound when *Xist* is activated in Eed^−/−^ Δ*Tsix* cells under 2i conditions, we performed ChIP analysis. We observed that despite activation of *Xist* in Eed^−/−^ Δ*Tsix* cells, Nanog and Oct4 binding to *Xist* intron 1 remained unchanged from the pattern in control cells (Fig. 3B, *P* = 0.54, *n* = 3; Fig. 3C, *P* = 0.19 and 0.50, *n* = 2). This observation that Oct4 and Nanog binding is not sufficient for repressing *Xist* in 2i, where *Rex1* and *Nanog* are homogenously expressed, is further consistent with previous reports that the binding site for Oct4 and Nanog in *Xist* intron 1 is dispensable for *Xist* regulation in embryos (22). Although a deletion of the binding site slightly skews the X chromosome for inactivation in female cells (21), we conclude that loss of pluripotency factor binding to *Xist* intron 1 does not explain the activation of *Xist* in Eed^−/−^ Δ*Tsix* cells.

### Ring1b is dispensable for *Tsix*-independent *Xist* repression.

**FIG 4:**
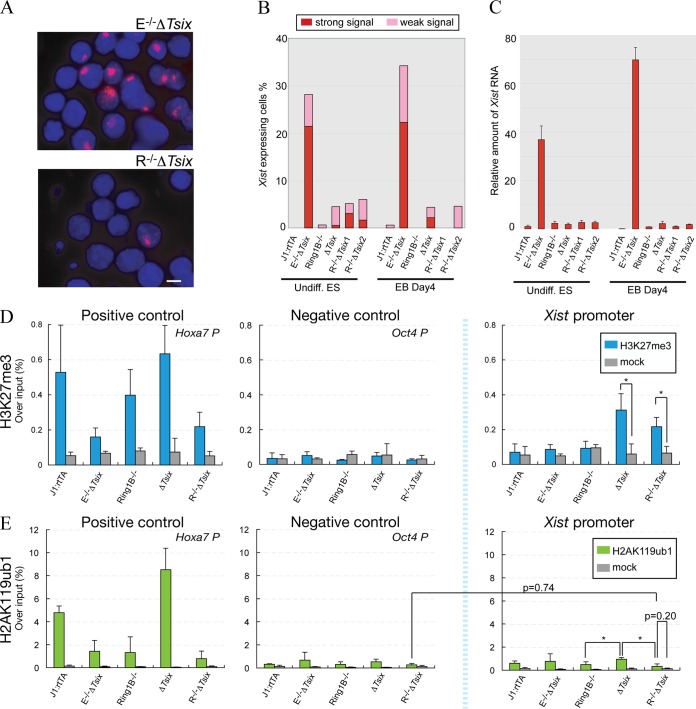
*Xist* expression is largely repressed in R^−/−^ Δ*Tsix* ES cells. (A) *Xist* RNA-FISH (red) in E^−/−^ Δ*Tsix* and R^−/−^ Δ*Tsix* cells differentiated for 4 days. Nuclei were counterstained with DAPI (blue). Bar, 10 μm. (B) Number of *Xist*-positive nuclei measured by *Xist* RNA-FISH (*n* > 150). (C) *Xist* expression (spliced product; exons 1 and 2) by qRT-PCR analysis is shown relative to undifferentiated J1:rtTA ES cells and normalized to *Gapdh*. (D and E) ChIP analysis of H3K27me3 (D) and H2AK119ub1 (E). *Hoxa7* and *Oct4* promoters were used as positive- and negative-control loci, respectively. Values are means and standard deviations from three independent experiments. Cells were cultured in ES medium with (undifferentiated) or without (differentiated) LIF. *, *P* < 0.05 (Student's *t* test).

PRC2-catalyzed H3K27me3 can act as a signal for recruitment of PRC1, consistent with observations of corecruitment of PRC1 and PRC2 to a large number of genes (45). PRC1 contains the RING finger domain proteins Ring1a and Ring1b and catalyzes monoubiquitination of histone H2A lysine 119 (H2AK119ub1) (46), which has been proposed to repress transcription by restraining poised RNA polymerase II (35). To investigate the potential function of PRC1 in *Xist* repression, we disrupted *Tsix* in ES cells that lack *Ring1b* (R^−/−^ Δ*Tsix*) (see Fig. S1 in the supplemental material). In contrast to Eed^−/−^ Δ*Tsix* ES cells, which showed activation of *Xist* (Fig. 4A to C), *Xist* remained repressed in R^−/−^ Δ*Tsix* ES cells (Fig. 4A to C). Although we could observe a few *Xist* clouds in R^−/−^ Δ*Tsix* ES cells (Fig. 4A and B), there was no significant difference between R^−/−^ Δ*Tsix* and Δ*Tsix* ES cells (Fig. 4B). Quantitative PCR confirmed that *Xist* remained repressed in R^−/−^ Δ*Tsix* ES cells (Fig. 4C). This observation demonstrated that *Ring1b* is not critical for *Tsix*-independent *Xist* repression.

ChIP analysis showed that H3K27me3 was enriched on the *Xist* promoter R^−/−^ Δ*Tsix* cells to a level comparable to that of Δ*Tsix* cells (Fig. 4D, *Xist* promoter [Δ*Tsix*, 5.27- ± 1.59-fold enrichment compared to the mock control {*P* < 0.05}; R^−/−^ Δ*Tsix*, 3.34- ± 0.81-fold enrichment {*P* < 0.05}). H2AK119ub1 was weakly enriched on the *Xist* promoter in Δ*Tsix* cells and furthermore was reduced in Ring1b^−/−^ ES cells (Fig. 4E) (1.95- ± 0.31-fold change [*P* < 0.05]) and R^−/−^ Δ*Tsix* cells (Fig. 4E) (2.89- ± 0.46-fold change [*P* < 0.05]). In Ring1b^−/−^ ES cells, a very small amount of H2AK119ub1 could still be observed (see Fig. S1G in the supplemental material) that was not significantly increased upon deletion of *Tsix* in R^−/−^ Δ*Tsix* cells (Fig. 4E, *Xist* promoter [2.48- ± 1.65-fold change {*P* = 0.20} relative to the mock control]). We cannot fully rule out the possibility that Ring1a catalyzes low levels of H2AK119ub1 in the absence of Ring1b in R^−/−^ Δ*Tsix* cells. However, in R^−/−^ Δ*Tsix* cells, H2AK119ub1 enrichment on the *Xist* promoter was similar to the *Oct4* promoter, which served as a negative control (Fig. 4E, compare R^−/−^ Δ*Tsix* data obtained with the *Xist* promoter to those obtained with the Oct4 promoter; 1.19- ± 0.79-fold change [*P* = 0.74]). Therefore, we conclude that establishment of H3K27me3 on the *Xist* promoter does not lead to efficient H2AK119ub1 recruitment, consistent with earlier results of low enrichment of H2AK119ub1 at the *Xist* promoter in serum-cultured ES cells (15). In addition, *Ring1b* and H2AK119ub1 appeared to be largely dispensable for *Xist* repression when *Tsix* was disrupted. Since H3K27me3 was still present at the *Xist* promoter, these observations pointed toward a PRC1-independent function of PRC2 in repressing the *Xist* promoter. This finding is surprising but consistent with observations of PRC1-independent PRC2 recruitment at a subset of genes in ES cells (45) as well as the idea of an evolutionarily older origin of PRC2, which is present in plants where PRC1 is not conserved (46).

### DNA methylation is dispensable for *Xist* repression by *Tsix*.

**FIG 5:**
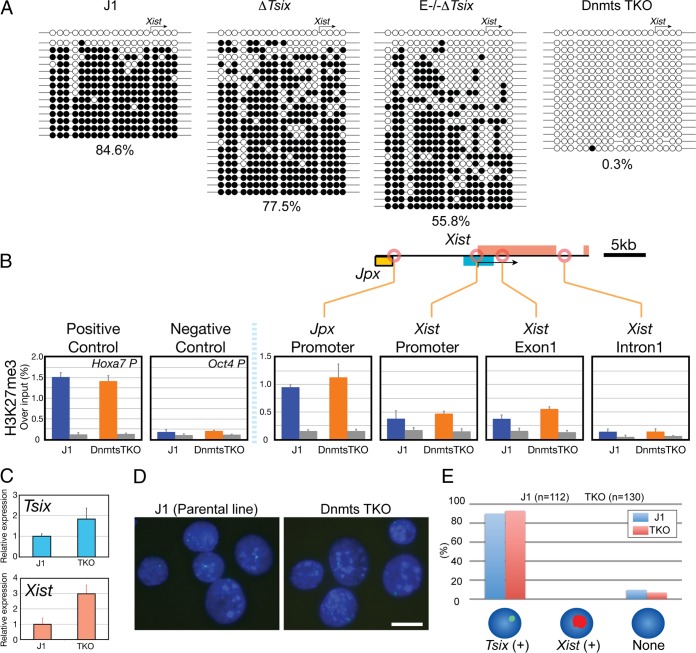
*Xist* expression is largely repressed in Dnmt TKO ES cells. (A) Cytosine DNA methylation was measured by bisulfite sequencing of the *Xist* promoter (filled circles, methylated; open circles, unmethylated). The percent methylation is given below the graphs. (B) ChIP analysis of H3K27me3 (*n* = 3). *Hoxa7* and *Oct4* promoters were used as positive- and negative-control loci, respectively. (C) qRT-PCR analysis for *Xist* and *Tsix* expression in Dnmt TKO and parental J1 ES cells. Expression relative to undifferentiated J1 ES cells was normalized to *Gapdh* (*n* = 2). (D) *Xist* (red) and *Tsix* (green) RNA-FISH in parental J1 (right) and Dnmt TKO (left) undifferentiated ES cells. Nuclei were counterstained by DAPI (blue). Bar, 10 μm. (E) Percent *Tsix*- or *Xist*-positive nuclei, revealed by RNA-FISH (*n* > 100).

Taken together, our results suggested that a repression mechanism based on PRC2-mediated H3K27me3 acts on genes within the *Xic* region. However, this mechanism is apparently disrupted at the *Xist* promoter by *Tsix*, which prevents the spreading of H3K27me3 from the hot spot over the promoter and gene body of *Xist*. Since previous studies have shown that *Tsix* is important for DNA methylation at the *Xist* promoter (10, 17, 36, 39, 47, 48) and DNA methylation is inversely correlated with Polycomb recruitment (49), we investigated whether DNA methylation might explain the mechanism *Tsix* uses for blocking H3K27me3 spreading. DNA methylation was present on the *Xist* promoter in the naive ES cells and decreased after *Tsix* disruption in Δ*Tsix* cells (Fig. 5A [J1, 84.6%; Δ*Tsix*, 77.5%]). This observation confirmed that *Tsix* contributed to DNA methylation on the *Xist* promoter in naive male ES cells, similar to earlier findings in serum-cultured ES cells. To analyze the function of DNA methylation, we used ES cells harboring combined mutations of all three DNA methyltransferases *Dnmt1*, *Dnmt3a*, and *Dnmt3b* (32). In these *Dnmt* triple-knockout (TKO) cells, DNA methylation at the *Xist* promoter was essentially absent (0.3%) (Fig. 5A). Despite the loss of DNA methylation, we observed that H3K27me3 remained unchanged and was excluded from the *Xist* promoter and gene body (Fig. 5B [Dnmt TKO], *Xist* promoter [0.47 ± 0.05], *Xist* exon 1 [0.55 ± 0.04], and *Xist* intron 1 [0.14 ± 0.05; values are relative to input values], and (Fig. 2B [Δ*Tsix*], *Xist* promoter [2.23 ± 0.73], *Xist* exon 1 [6.01 ± 2.23], and *Xist* intron 1 [1.32 ± 0.26]). We further observed that *Tsix* transcription was slightly increased in Dnmt TKO cells (Fig. 5C, *Tsix*) (1.87- ± 0.66-fold change). In Dnmt TKO cells, DNA methylation was lost (Fig. 5A) and spreading of H3K27me3 from the hot spot to the *Xist* promoter was blocked by *Tsix* (Fig. 5B), leaving the *Xist* promoter unaffected by these two repressive modifications. This prompted us to investigate whether, in this situation, reactivation of *Xist* could be observed, similar to that in Eed^−/−^ Δ*Tsix* cells, in which DNA methylation at the *Xist* promoter was also significantly decreased (Fig. 5A, E^−/−^ Δ*Tsix* [55.8%]). We observed only a moderate upregulation of *Xist* in Dnmt TKO cells (Fig. 5C, *Xist*) (3.01 ± 0.33), comparable to the increased *Xist* expression in single Eed^−/−^ and Δ*Tsix* mutant ES cells (Fig. 1C). Furthermore, no *Xist* clusters were observed in Dnmt TKO cells by *Xist* RNA-FISH (Fig. 5D and E), demonstrating that *Tsix* represses *Xist* through additional mechanisms, when neither DNA methylation nor H3K27me3 is present at the *Xist* promoter.

### Tsix recruits H3K36me3 to the *Xist* promoter.

**FIG 6:**
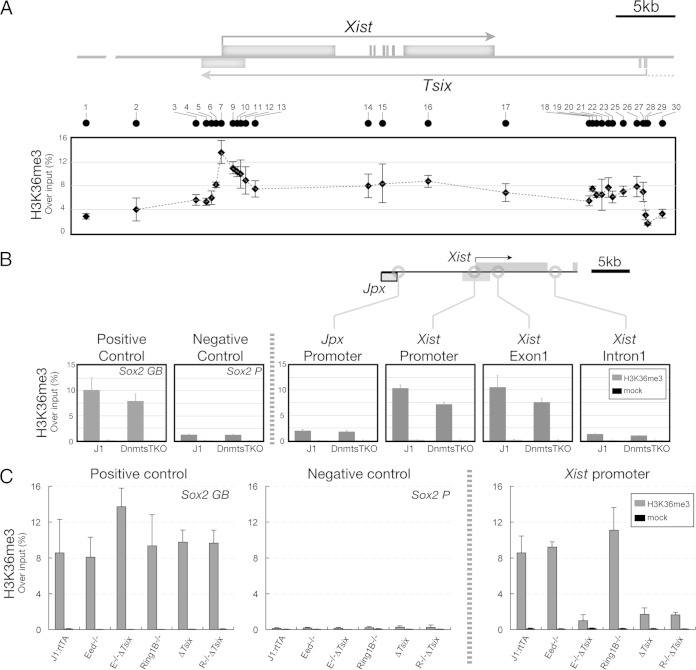
H3K36me3 is accompanied by *Tsix* transcription. (A) ChIP analysis of H3K36me3 in J1 cells covering the entire transcription unit of *Tsix* (39). (B and C) ChIP analysis of H3K36me3 in Dnmt TKO cells and its parental line, J1 (B), and J1:rtTA, Eed^−/−^, E^−/−^ Δ*Tsix*, Ring1b^−/−^, Δ*Tsix*, and R^−/−^ Δ*Tsix* cells (C). The *Sox2* gene body and its promoter were used as positive- and negative-control loci, respectively. Data are means and standard deviations from three independent experiments.

To identify such additional mechanisms, we considered histone marks associated with coding and noncoding transcription. H3K36me3 is correlated with transcriptional elongation and repression of inappropriate initiation of transcription within the gene body (50–54). We performed ChIP to investigate whether H3K36me3 was established by *Tsix* transcription over the *Xist* gene body and promoter (39). We detected a strong enrichment of H3K36me3 over the entire *Xist* locus that, importantly, also included the *Xist* promoter (Fig. 6A). The enrichment of H3K36me3 over *Xist* is also observed in genome-wide data sets of serum-cultured ES cells (see Fig. S2 in the supplemental material). We observed H3K36me3 in both J1 and Dnmt TKO cells at the *Xist* promoter (Fig. 6B, *Xist* promoter [J1, 10.35% ± 0.15% of input; Dnmt TKO, 7.17% ± 0.09%]) and exon 1 (Fig. 6B [J1, 10.52% ± 2.34%; Dnmt TKO, 7.57% ± 0.69%]). H3K36me3 was not detected upstream (Fig. 6B, *Jpx* promoter [J1, 1.98% ± 0.24% of input; Dnmt TKO, 1.81% ± 0.16%]) or far downstream of *Xist* (Fig. 6B, *Xist* intron 1 [J1, 1.98% ± 0.24% of input; Dnmt TKO, 1.00% ± 0.06%]), where we observed levels of H3K36me3 that are comparable to the negative-control *Sox2* promoter (Fig. 6B [J1, 1.27% ± 0.10% of input; Dnmt TKO, 1.24% ± 0.11%]). An enrichment of H3K36me3 on the *Xist* promoter was also observed in J1:rtTA, Eed^−/−^, and Ring1b^−/−^ ES cells (Fig. 6C, *Xist* promoter [J1:rtTA, 8.55% ± 1.89% of input; Eed^−/−^, 9.20% ± 0.59%; Ring1b^−/−^, 11.09% ± 2.53%]), demonstrating that H3K36me3 is enriched at the *Xist* promoter and does not require PRC function. Importantly, H3K36me3 was strongly reduced to near background levels in ES cells that lack *Tsix* (Fig. 6C, *Xist* promoter [Δ*Tsix*, 1.69% ± 0.73%; E^−/−^ Δ*Tsix*, 0.99% ± 0.67%; R^−/−^ Δ*Tsix*, 1.63% ± 0.31%]). Taken together, these findings show that *Tsix* transcription is required for establishing H3K36me3 at the *Xist* promoter.

### H3K36me3 contributes to repression of *Xist* transcription.

**FIG 7:**
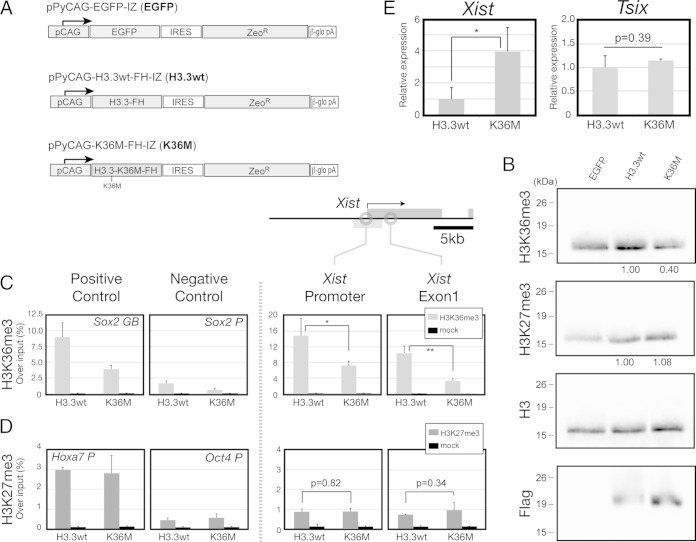
H3K36me3 is functionally involved in *Xist* repression. (A) Schematic representation of expression constructs. (B) Western blot analysis of histone modifications H3K36me3 and H3K27me3 in J1 cells expressing the indicated transgenes. H3 was used as a loading control, and Flag was used to confirm expression of a Flag-tagged transgene. The relative amount of histone modification, with the amount of H3.3wt set to 1 and normalized to histone H3, is given beneath the lanes in the top two panels. (C and D) ChIP analysis of H3K36me3 (C) and H3K27me3 (D) in J1 cells expressing H3.3wt and K36M transgenes. The *Sox2* gene body and its promoter for H3K36me3 and the *Hoxa7* and *Oct4* promoters for H3K27me3 were used as positive- and negative-control loci, respectively. Data are means and standard deviations from three independent experiments. (E) qRT-PCR analysis for *Xist* and *Tsix* expression in H3.3wt-expressing (H3.3wt) and K36M-expressing (K36M) J1 ES cells. Expression relative to H3.3wt-expressing J1 ES cells normalized to *Gapdh* (*n* = 3) is shown. *, *P* < 0.05; **, *P* < 0.01 (Student's *t* test).

H3K36me3 recruitment on the *Xist* promoter via *Tsix* transcription prompted us to investigate whether it could exert a repressive function on the *Xist* promoter in *cis*. In mammals, to date, at least eight histone H3 lysine K36 methyltransferases (H3K36 HKMTs) have been reported, including those encoded by the *NSD1*, *NSD2*, *NSD3*, *SETD2*, *SETD3*, *ASH1L*, *SETMAR*, and *SMYD2* genes (55). The number of H3K36 HKMTs and their potential redundancy make it difficult to eliminate H3K36 methylation. To address this issue comprehensively and also to consider potential cell viability issues, we selected two complementary strategies. First, we aimed to disrupt H3K36 methylation through expression of a mutant histone protein, H3.3-K36M (K36M), that has a substitution of methionine for lysine at position 36. This mutant protein has been previously shown to reduce endogenous K36 methylation through binding and sequestering H3K36 HKMTs (56). We established stable transgenic J1 ES cell pools that express wild-type and mutated histone H3.3 proteins (Fig. 7A). Anti-Flag immunoblotting (Fig. 7B, Flag) and long exposure of anti-H3 (see Fig. S3 in the supplemental material) confirmed the expression of H3.3wt and H3.3-K36M. The total amount of endogenous H3K36me3 was only slightly increased by transgenic expression of H3.3wt (Fig. 7B, H3K36me3) (1.22-fold change compared to H3.3wt with EGFP cells, normalized to H3). In contrast, in K36M-expressing cells, H3K36me3 was decreased to approximately half the level of control cells (Fig. 7B, H3K36me3) (0.40-fold change for K36M compared with H3.3wt, normalized to H3). Importantly, H3K36me3 was also reduced over the *Xist* promoter (Fig. 7C) (0.48- ± 0.07-fold change for the K36M mutant compared with H3.3wt [*P* < 0.05]) and *Xist* exon 1 (Fig. 7C) (0.31- ± 0.07-fold change for the K36M mutant compared with H3.3wt [*P* < 0.01]). To test for specificity, we also measured H3K27me3, which remained unchanged globally (Fig. 7B, H3K27me3) (1.08-fold change for the K36M mutant compared with H3.3wt, normalized to H3) and locally on the *Xist* promoter (Fig. 7D) (1.04- ± 0.18-fold change for the K36M mutant compared with H3.3wt [*P* = 0.82]) and *Xist* exon 1 (Fig. 7D) (1.35- ± 0.56-fold change for the K36M mutant compared with H3.3wt [*P* = 0.34]). We did not measure an increase in the number of *Xist* clusters by RNA-FISH (see Fig. S4 in the supplemental material), but we did observe a significant derepression of *Xist* in K36M-overexpressing cells by qRT-PCR (Fig. 7E, *Xist*) (3.95- ± 1.49-fold change [*P* < 0.05]), whereas the expression of *Tsix* remained unchanged (Fig. 7E, *Tsix*) (1.15- ± 0.04-fold change [*P* = 0.39]). To obtain independent evidence by a second method, we aimed to reduce H3K36me3 using an RNA interference-mediated depletion of *Setd2*. The product of *Setd2* is considered a major H3K36 methylase responsible for H3K36me3 (55). We observed a significant derepression of *Xist* in J1 ES cells after RNA interference-mediated depletion of *Setd2* (see Fig. S5 in the supplemental material). Importantly, under our experimental conditions, the expression of *Tsix* remained unaffected. Taken together, our data suggest that H3K36me3 is established in a *Tsix*-dependent manner over the *Xist* promoter and contributes to a repressive effect on the *Xist* promoter.

## DISCUSSION

In our study, we addressed the function of chromatin modifications regulating the *Xist* promoter. Our results suggest two independent repressive mechanisms: one is mediated by *Tsix* transcription, and a second and independent mechanism is mediated by PRC2. Although this is a relatively simple scenario, which accurately predicts the derepression of *Xist* when both repressive mechanisms are impaired in *Tsix*- and *Eed*-deficient cells, further insights into the molecular interactions are needed. Here, we dissected the functions of several known and one novel chromatin modification in this system. Combined disruption of *Tsix* and *Eed* caused activation of *Xist* in naive ES cells. Notably, *Xist* was activated, although the binding of Nanog and Oct4 in *Xist* intron 1 was preserved, demonstrating that Nanog and Oct4 binding is not sufficient for *Xist* repression. This observation is consistent with a recent report showing that the intron 1 binding site is not essential for *Xist* repression (21, 22). However, our data do not rule out the possibility that Oct4 and Nanog repress *Xist* through recruitment of PRC2. Indeed, a previous study reported a synergistic effect between the loss of the Oct4 binding site in *Xist* intron 1 and *Tsix* on *Xist* activation (57). Furthermore, Oct4 could possess independent modulatory functions in the repression of *Xist* that are not critical in male cells.

In our system, repression of *Xist* by *Tsix* required neither DNA methylation nor Polycomb recruitment, suggesting that additional mechanisms exist by which *Tsix* exerts its repressive function. We find that H3K36me3 is recruited to the *Xist* promoter in naive as well as serum-cultured ES cells. We show that H3K36me3 recruitment at the *Xist* promoter and gene body depends on *Tsix* transcription but requires neither the PRC complex proteins Ring1b and Eed nor DNA methylation. H3K36me3 is associated with transcriptional elongation and a function in repression of inappropriate transcription (50–54). To address the function of H3K36me3 in repressing *Xist*, we performed two experiments aiming at reducing H3K36me3 globally. The first approach is based on a mutated form of histone H3.3 that is able to sequester histone methylases specifically (56). Expression of a histone H3.3 carrying a substitution of methionine for lysine at position 36 led to a reduction of endogenous H3K36me3 to half the amount observed in control cells. Reduced H3K36me3 caused a marked derepression of *Xist* but had little effect on *Tsix* and a control gene. Our second and independent attempt at reducing H3K36me3 was based on RNA interference-mediated depletion of *Setd2. Setd2* is considered the major histone methyltransferase catalyzing H3K36me3. This approach was less efficient in reducing H3K36me3 but also resulted in *Xist* upregulation. Taken together, our experiments support the view that H3K36me3 recruitment contributes to *Xist* repression. However, we were not able to eliminate H3K36me3 entirely and thus may have observed a partial effect on *Xist* expression. In addition, depletion of H3K36me3 might also have indirect effects through perturbation of transcription units of other genes. We did not observe *Xist* clusters after depletion of H3K36me3 by RNA-FISH, despite the fact that H3K27me3 and H3K36me3 were reduced at the *Xist* promoter, suggesting that additional mechanisms exist through which *Tsix* represses *Xist*.

In the absence of *Tsix*, H3K27me3 is recruited to the *Xist* promoter through PRC2. H3K27me3 apparently spreads from a hot spot that is located 5′ to *Xist*. When *Eed* is mutated, H3K27me3 is lost and *Xist* is activated. We find that loss of H3K27me3 enrichment also leads to derepression of the *Ftx*, *Jpx*, *Cnbp2*, and *Xpct* genes, suggesting that multiple genes within the *Xic* are repressed by PRC2. The expression of *Ftx* (25) and *Jpx* (23) is elevated in differentiating female ES cells and correlates with *Xist* activation. Currently, the function of *Cnbp2* in XCI is unknown, and overexpression of *Xpct* does not lead to *Xist* expression (58).

Notably, H3K27me3 at the *Xist* promoter does not efficiently recruit H2AK119ub1. H2AK119ub1 is reduced in *Ring1b*-deficient cells to near background levels, but simultaneous loss of *Ring1b* and *Tsix* did not result in activation of *Xist*. This observation could suggest that at the *Xist* promoter H3K27me3 exerts a repressive function that is largely independent of H2AK119ub1. A PRC1-independent function of PRC2 is presently not regarded as a prominent silencing mechanism. However, genes have been identified in ES cells that are targets of PRC2 but not PRC1, suggesting that this mode of regulation is not entirely specific to the *Xist* gene promoter (45).

In cells carrying an intact *Tsix* gene, H3K27me3 appears to spread from a hot spot upstream of *Xist* over several genes within the *Xic* region but is prevented from entering the *Xist* locus. We aimed to clarify whether known chromatin modifications can explain how *Tsix* prevents the spreading of H3K27me3. Previous reports have shown that DNA methylation is recruited by *Tsix* to the *Xist* promoter (10, 17, 36, 39, 47, 48). Notably, DNA methylation and H3K27me3 are inversely correlated in undifferentiated ES cells (49). Using ES cells that lack DNA methylation, we demonstrated that DNA methylation is not required for restricting the spreading of H3K27me3. H3K27me3 also remained excluded from the *Xist* promoter after depletion of H3K36me3. This observation suggests that H3K36me3 is not critical for restricting spreading of H3K27me3 over the *Xist* promoter. However, we cannot rule out the possibility that we have not been able to reduce H3K36me3 to low enough levels. Blocking of H3K27me3 spreading by *Tsix* might involve functional redundancies between H3K36me3 and DNA methylation. Alternatively, additional, as-yet-unidentified mechanisms might exist.

Our data implicate H3K36me3 as a chromatin modification that is established cotranscriptionally by *Tsix* over the *Xist* promoter and contributes to *Xist* repression. This modification can now be considered in future analyses of *Xist* regulation and will facilitate progress in understanding the chromatin-based mechanisms that contribute to the initiation of XCI. It will also be interesting to see if H3K36me3 is also relevant for gene regulation by noncoding transcription at other gene loci.

## Supplementary Material

Supplemental material

## References

[1] Schulz EG, Heard E 2013 Role and control of X chromosome dosage in mammalian development. Curr Opin Genet Dev 23:109–115. doi:10.1016/j.gde.2013.01.008. 23465885

[2] Lee JT, Davidow LS, Warshawsky D 1999 Tsix, a gene antisense to Xist at the X-inactivation centre. Nat Genet 21:400–404. doi:10.1038/7734. 10192391

[3] Donohoe ME, Silva SS, Pinter SF, Xu N, Lee JT 2009 The pluripotency factor Oct4 interacts with Ctcf and also controls X-chromosome pairing and counting. Nature 460:128–132. doi:10.1038/nature08098. 19536159 PMC3057664

[4] Gontan C, Achame EM, Demmers J, Barakat TS, Rentmeester E, van IJken W, Grootegoed JA, Gribnau J 2012 RNF12 initiates X-chromosome inactivation by targeting REX1 for degradation. Nature 485:386–390. doi:10.1038/nature11070. 22596162

[5] Guyochin A, Maenner S, Chu ET, Hentati A, Attia M, Avner P, Clerc P 2014 Live cell imaging of the nascent inactive X chromosome during the early differentiation process of naive ES cells towards epiblast stem cells. PLoS One 9:e116109. doi:10.1371/journal.pone.0116109. 25546018 PMC4278889

[6] Lee JT, Lu N 1999 Targeted mutagenesis of Tsix leads to nonrandom X inactivation. Cell 99:47–57. doi:10.1016/S0092-8674(00)80061-6. 10520993

[7] Luikenhuis S, Wutz A, Jaenisch R 2001 Antisense transcription through the Xist locus mediates Tsix function in embryonic stem cells. Mol Cell Biol 21:8512–8520. doi:10.1128/MCB.21.24.8512-8520.2001. 11713286 PMC100014

[8] Sado T, Li E, Sasaki H 2002 Effect of TSIX disruption on XIST expression in male ES cells. Cytogenet Genome Res 99:115–118. doi:10.1159/000071582. 12900553

[9] Vigneau S, Augui S, Navarro P, Avner P, Clerc P 2006 An essential role for the DXPas34 tandem repeat and Tsix transcription in the counting process of X chromosome inactivation. Proc Natl Acad Sci U S A 103:7390–7395. doi:10.1073/pnas.0602381103. 16648248 PMC1464350

[10] Ohhata T, Senner CE, Hemberger M, Wutz A 2011 Lineage-specific function of the noncoding Tsix RNA for Xist repression and Xi reactivation in mice. Genes Dev 25:1702–1715. doi:10.1101/gad.16997911. 21852535 PMC3165935

[11] Lee JT 2000 Disruption of imprinted X inactivation by parent-of-origin effects at Tsix. Cell 103:17–27. doi:10.1016/S0092-8674(00)00101-X. 11051544

[12] Sado T, Wang Z, Sasaki H, Li E 2001 Regulation of imprinted X-chromosome inactivation in mice by Tsix. Development 128:1275–1286. 11262229 10.1242/dev.128.8.1275

[13] Ohhata T, Hoki Y, Sasaki H, Sado T 2006 Tsix-deficient X chromosome does not undergo inactivation in the embryonic lineage in males: implications for Tsix-independent silencing of Xist. Cytogenet Genome Res 113:345–349. doi:10.1159/000090851. 16575199

[14] Migeon BR 2003 Is Tsix repression of Xist specific to mouse? Nat Genet 33:337. doi:10.1038/ng0303-337a (Reply, 33:337–338, doi:10.1038/ng0303-337a.) 12610550

[15] Shibata S, Yokota T, Wutz A 2008 Synergy of Eed and Tsix in the repression of Xist gene and X-chromosome inactivation. EMBO J 27:1816–1826. doi:10.1038/emboj.2008.110. 18511907 PMC2486422

[16] Montgomery ND, Yee D, Chen A, Kalantry S, Chamberlain SJ, Otte AP, Magnuson T 2005 The murine polycomb group protein Eed is required for global histone H3 lysine-27 methylation. Curr Biol 15:942–947. doi:10.1016/j.cub.2005.04.051. 15916951

[17] Sun BK, Deaton AM, Lee JT 2006 A transient heterochromatic state in Xist preempts X inactivation choice without RNA stabilization. Mol Cell 21:617–628. doi:10.1016/j.molcel.2006.01.028. 16507360

[18] Jonkers I, Barakat TS, Achame EM, Monkhorst K, Kenter A, Rentmeester E, Grosveld F, Grootegoed JA, Gribnau J 2009 RNF12 is an X-encoded dose-dependent activator of X chromosome inactivation. Cell 139:999–1011. doi:10.1016/j.cell.2009.10.034. 19945382

[19] Navarro P, Chambers I, Karwacki-Neisius V, Chureau C, Morey C, Rougeulle C, Avner P 2008 Molecular coupling of Xist regulation and pluripotency. Science 321:1693–1695. doi:10.1126/science.1160952. 18802003

[20] Navarro P, Oldfield A, Legoupi J, Festuccia N, Dubois A, Attia M, Schoorlemmer J, Rougeulle C, Chambers I, Avner P 2010 Molecular coupling of Tsix regulation and pluripotency. Nature 468:457–460. doi:10.1038/nature09496. 21085182

[21] Barakat TS, Gunhanlar N, Pardo CG, Achame EM, Ghazvini M, Boers R, Kenter A, Rentmeester E, Grootegoed JA, Gribnau J 2011 RNF12 activates Xist and is essential for X chromosome inactivation. PLoS Genet 7:e1002001. doi:10.1371/journal.pgen.1002001. 21298085 PMC3029249

[22] Minkovsky A, Barakat TS, Sellami N, Chin MH, Gunhanlar N, Gribnau J, Plath K 2013 The pluripotency factor-bound intron 1 of Xist is dispensable for X chromosome inactivation and reactivation in vitro and in vivo. Cell Rep 3:905–918. doi:10.1016/j.celrep.2013.02.018. 23523354 PMC3615097

[23] Tian D, Sun S, Lee JT 2010 The long noncoding RNA, Jpx, is a molecular switch for X chromosome inactivation. Cell 143:390–403. doi:10.1016/j.cell.2010.09.049. 21029862 PMC2994261

[24] Sun S, Del Rosario BC, Szanto A, Ogawa Y, Jeon Y, Lee JT 2013 Jpx RNA activates Xist by evicting CTCF. Cell 153:1537–1551. doi:10.1016/j.cell.2013.05.028. 23791181 PMC3777401

[25] Chureau C, Chantalat S, Romito A, Galvani A, Duret L, Avner P, Rougeulle C 2011 Ftx is a non-coding RNA which affects Xist expression and chromatin structure within the X-inactivation center region. Hum Mol Genet 20:705–718. doi:10.1093/hmg/ddq516. 21118898

[26] Soma M, Fujihara Y, Okabe M, Ishino F, Kobayashi S 2014 Ftx is dispensable for imprinted X-chromosome inactivation in preimplantation mouse embryos. Sci Rep 4:5181. doi:10.1038/srep05181. 24899465 PMC5381492

[27] Augui S, Filion GJ, Huart S, Nora E, Guggiari M, Maresca M, Stewart AF, Heard E 2007 Sensing X chromosome pairs before X inactivation via a novel X-pairing region of the Xic. Science 318:1632–1636. doi:10.1126/science.1149420. 18063799

[28] Bacher CP, Guggiari M, Brors B, Augui S, Clerc P, Avner P, Eils R, Heard E 2006 Transient colocalization of X-inactivation centres accompanies the initiation of X inactivation. Nat Cell Biol 8:293–299. doi:10.1038/ncb1365. 16434960

[29] Xu N, Tsai CL, Lee JT 2006 Transient homologous chromosome pairing marks the onset of X inactivation. Science 311:1149–1152. doi:10.1126/science.1122984. 16424298

[30] Wutz A, Jaenisch R 2000 A shift from reversible to irreversible X inactivation is triggered during ES cell differentiation. Mol Cell 5:695–705. doi:10.1016/S1097-2765(00)80248-8. 10882105

[31] Ying QL, Wray J, Nichols J, Batlle-Morera L, Doble B, Woodgett J, Cohen P, Smith A 2008 The ground state of embryonic stem cell self-renewal. Nature 453:519–523. doi:10.1038/nature06968. 18497825 PMC5328678

[32] Tsumura A, Hayakawa T, Kumaki Y, Takebayashi S, Sakaue M, Matsuoka C, Shimotohno K, Ishikawa F, Li E, Ueda HR, Nakayama J, Okano M 2006 Maintenance of self-renewal ability of mouse embryonic stem cells in the absence of DNA methyltransferases Dnmt1, Dnmt3a and Dnmt3b. Genes Cells 11:805–814. doi:10.1111/j.1365-2443.2006.00984.x. 16824199

[33] Leeb M, Wutz A 2007 Ring1B is crucial for the regulation of developmental control genes and PRC1 proteins but not X inactivation in embryonic cells. J Cell Biol 178:219–229. doi:10.1083/jcb.200612127. 17620408 PMC2064442

[34] Niwa H, Toyooka Y, Shimosato D, Strumpf D, Takahashi K, Yagi R, Rossant J 2005 Interaction between Oct3/4 and Cdx2 determines trophectoderm differentiation. Cell 123:917–929. doi:10.1016/j.cell.2005.08.040. 16325584

[35] Stock JK, Giadrossi S, Casanova M, Brookes E, Vidal M, Koseki H, Brockdorff N, Fisher AG, Pombo A 2007 Ring1-mediated ubiquitination of H2A restrains poised RNA polymerase II at bivalent genes in mouse ES cells. Nat Cell Biol 9:1428–1435. doi:10.1038/ncb1663. 18037880

[36] Sado T, Hoki Y, Sasaki H 2005 Tsix silences Xist through modification of chromatin structure. Dev Cell 9:159–165. doi:10.1016/j.devcel.2005.05.015. 15992549

[37] Marks H, Kalkan T, Menafra R, Denissov S, Jones K, Hofemeister H, Nichols J, Kranz A, Stewart AF, Smith A, Stunnenberg HG 2012 The transcriptional and epigenomic foundations of ground state pluripotency. Cell 149:590–604. doi:10.1016/j.cell.2012.03.026. 22541430 PMC3398752

[38] Hayashi-Takanaka Y, Yamagata K, Wakayama T, Stasevich TJ, Kainuma T, Tsurimoto T, Tachibana M, Shinkai Y, Kurumizaka H, Nozaki N, Kimura H 2011 Tracking epigenetic histone modifications in single cells using Fab-based live endogenous modification labeling. Nucleic Acids Res 39:6475–6488. doi:10.1093/nar/gkr343. 21576221 PMC3159477

[39] Navarro P, Page DR, Avner P, Rougeulle C 2006 Tsix-mediated epigenetic switch of a CTCF-flanked region of the Xist promoter determines the Xist transcription program. Genes Dev 20:2787–2792. doi:10.1101/gad.389006. 17043308 PMC1619945

[40] Nagano T, Mitchell JA, Sanz LA, Pauler FM, Ferguson-Smith AC, Feil R, Fraser P 2008 The Air noncoding RNA epigenetically silences transcription by targeting G9a to chromatin. Science 322:1717–1720. doi:10.1126/science.1163802. 18988810

[41] Atsuta T, Fujimura S, Moriya H, Vidal M, Akasaka T, Koseki H 2001 Production of monoclonal antibodies against mammalian Ring1B proteins. Hybridoma 20:43–46. doi:10.1089/027245701300060427. 11289226

[42] Leitch HG, McEwen KR, Turp A, Encheva V, Carroll T, Grabole N, Mansfield W, Nashun B, Knezovich JG, Smith A, Surani MA, Hajkova P 2013 Naive pluripotency is associated with global DNA hypomethylation. Nat Struct Mol Biol 20:311–316. doi:10.1038/nsmb.2510. 23416945 PMC3591483

[43] Rougeulle C, Chaumeil J, Sarma K, Allis CD, Reinberg D, Avner P, Heard E 2004 Differential histone H3 Lys-9 and Lys-27 methylation profiles on the X chromosome. Mol Cell Biol 24:5475–5484. doi:10.1128/MCB.24.12.5475-5484.2004. 15169908 PMC419884

[44] Navarro P, Chantalat S, Foglio M, Chureau C, Vigneau S, Clerc P, Avner P, Rougeulle C 2009 A role for non-coding Tsix transcription in partitioning chromatin domains within the mouse X-inactivation centre. Epigenet Chromatin 2:8. doi:10.1186/1756-8935-2-8. PMC2720958 19615107

[45] Ku M, Koche RP, Rheinbay E, Mendenhall EM, Endoh M, Mikkelsen TS, Presser A, Nusbaum C, Xie X, Chi AS, Adli M, Kasif S, Ptaszek LM, Cowan CA, Lander ES, Koseki H, Bernstein BE 2008 Genomewide analysis of PRC1 and PRC2 occupancy identifies two classes of bivalent domains. PLoS Genet 4:e1000242. doi:10.1371/journal.pgen.1000242. 18974828 PMC2567431

[46] Schwartz YB, Pirrotta V 2007 Polycomb silencing mechanisms and the management of genomic programmes. Nat Rev Genet 8:9–22. doi:10.1038/nrg1981. 17173055

[47] Nesterova TB, Popova BC, Cobb BS, Norton S, Senner CE, Tang YA, Spruce T, Rodriguez TA, Sado T, Merkenschlager M, Brockdorff N 2008 Dicer regulates Xist promoter methylation in ES cells indirectly through transcriptional control of Dnmt3a. Epigenet Chromatin 1:2. doi:10.1186/1756-8935-1-2. PMC2577046 19014663

[48] Ohhata T, Hoki Y, Sasaki H, Sado T 2008 Crucial role of antisense transcription across the Xist promoter in Tsix-mediated Xist chromatin modification. Development 135:227–235. 18057104 10.1242/dev.008490

[49] Hagarman JA, Motley MP, Kristjansdottir K, Soloway PD 2013 Coordinate regulation of DNA methylation and H3K27me3 in mouse embryonic stem cells. PLoS One 8:e53880. doi:10.1371/journal.pone.0053880. 23326524 PMC3543269

[50] Carrozza MJ, Li B, Florens L, Suganuma T, Swanson SK, Lee KK, Shia WJ, Anderson S, Yates J, Washburn MP, Workman JL 2005 Histone H3 methylation by Set2 directs deacetylation of coding regions by Rpd3S to suppress spurious intragenic transcription. Cell 123:581–592. doi:10.1016/j.cell.2005.10.023. 16286007

[51] Joshi AA, Struhl K 2005 Eaf3 chromodomain interaction with methylated H3-K36 links histone deacetylation to Pol II elongation. Mol Cell 20:971–978. doi:10.1016/j.molcel.2005.11.021. 16364921

[52] Keogh MC, Kurdistani SK, Morris SA, Ahn SH, Podolny V, Collins SR, Schuldiner M, Chin K, Punna T, Thompson NJ, Boone C, Emili A, Weissman JS, Hughes TR, Strahl BD, Grunstein M, Greenblatt JF, Buratowski S, Krogan NJ 2005 Cotranscriptional set2 methylation of histone H3 lysine 36 recruits a repressive Rpd3 complex. Cell 123:593–605. doi:10.1016/j.cell.2005.10.025. 16286008

[53] Mikkelsen TS, Ku M, Jaffe DB, Issac B, Lieberman E, Giannoukos G, Alvarez P, Brockman W, Kim TK, Koche RP, Lee W, Mendenhall E, O'Donovan A, Presser A, Russ C, Xie X, Meissner A, Wernig M, Jaenisch R, Nusbaum C, Lander ES, Bernstein BE 2007 Genome-wide maps of chromatin state in pluripotent and lineage-committed cells. Nature 448:553–560. doi:10.1038/nature06008. 17603471 PMC2921165

[54] Nimura K, Ura K, Shiratori H, Ikawa M, Okabe M, Schwartz RJ, Kaneda Y 2009 A histone H3 lysine 36 trimethyltransferase links Nkx2-5 to Wolf-Hirschhorn syndrome. Nature 460:287–291. doi:10.1038/nature08086. 19483677

[55] Wagner EJ, Carpenter PB 2012 Understanding the language of Lys36 methylation at histone H3. Nat Rev Mol Cell Biol 13:115–126. doi:10.1038/nrm3274. 22266761 PMC3969746

[56] Lewis PW, Muller MM, Koletsky MS, Cordero F, Lin S, Banaszynski LA, Garcia BA, Muir TW, Becher OJ, Allis CD 2013 Inhibition of PRC2 activity by a gain-of-function H3 mutation found in pediatric glioblastoma. Science 340:857–861. doi:10.1126/science.1232245. 23539183 PMC3951439

[57] Nesterova TB, Senner CE, Schneider J, Alcayna-Stevens T, Tattermusch A, Hemberger M, Brockdorff N 2011 Pluripotency factor binding and Tsix expression act synergistically to repress Xist in undifferentiated embryonic stem cells. Epigenetics Chromatin 4:17. doi:10.1186/1756-8935-4-17. 21982142 PMC3197471

[58] Sun S, Fukue Y, Nolen L, Sadreyev R, Lee JT 2010 Characterization of Xpr (Xpct) reveals instability but no effects on X-chromosome pairing or Xist expression. Transcription 1:46–56. doi:10.4161/trns.1.1.12401. 21327163 PMC3035190

