# Supplementary material for: Histone H3 Lysine 36 Trimethylation Is Established over the Xist Promoter by Antisense Tsix Transcription and Contributes to Repressing Xist Expression
Source: Mol Cell Biol. 2015 Oct 16;35(22):3909–20. doi: 10.1128/MCB.00561-15 (PMC4609750; doi:10.1128/MCB.00561-15)
Supplement: Supplemental material [file supp_35_22_3909__index.html]

Histone H3 Lysine 36 Trimethylation Is Established over the Xist Promoter by Antisense Tsix Transcription and Contributes to Repressing Xist Expression — Supplemental material 

# Histone H3 Lysine 36 Trimethylation Is Established over the *Xist* Promoter by Antisense *Tsix* Transcription and Contributes to Repressing *Xist* Expression

## Supplemental material

- Supplemental file 1 -

  Supplemental text; Fig. S1 (Generation and characterization of Δ*Tsix* male ES cells), S2 (H3K36me3 installation along with *Tsix* transcription), S3 (Confirmation of H3.3wt and H3.3K36M expression), S4 (*Xist* accumulation in H3.3K36M-expressing cells), and S5 (*Xist* derepression in Setd2 knockdown cells); and Table S1 (PCR primer sequences)

  PDF, 368K
