# Supplementary material for: Histone H3 Lysine 36 Trimethylation Is Established over the Xist Promoter by Antisense Tsix Transcription and Contributes to Repressing Xist Expression
Source: Mol Cell Biol. 2015 Oct 16;35(22):3909–20. doi: 10.1128/MCB.00561-15 (PMC4609750; doi:10.1128/MCB.00561-15)
Supplement: Supplemental material [file MCB.00561-15_zmb999101013so1.pdf]

Supplemental material

Histone H3 lysine 36 tri-methylation is established over the *Xist* promoter by antisense *Tsix* transcription and contributes to repressing *Xist* expression

Tatsuya Ohhata[1,2+], Mika Matsumoto[1], Martin Leeb[2], Shinwa Shibata[4], Satoshi Sakai[1]

Kyoko Kitagawa[1], Hiroyuki Niida[1], Masatoshi Kitagawa[1+] and Anton Wutz[2,3+]

[1]Hamamatsu University School of Medicine, Hamamatsu, 431-3102, Japan
[2]WT and MRC Stem Cell Institute, University of Cambridge, CB2 1QR, UK
[3]Institute of Molecular Health Sciences, ETH, Zurich, 8006, Switzerland.
[4]Pharmaceuticals and medical devices agency, Tokyo, 100-0013, Japan

[+]Corresponding author
Tel: +41(0)446330848
Fax: +41(0)446331726
E-mail: awutz@ethz.ch

Tel/Fax: +81 53 435 2322
E-mail: kitamasa@hama-med.ac.jp
E-mail: ohhata@hama-med.ac.jp

Running title: *Xist* repression by *Tsix* induced H3K36me3

Supplemental Materials and Methods

Constructs

For generating the TST targeting vector, a 9.2kb *Sma*I-*Apa*I genomic fragment containing a part of *Tsix* exon2 and exon3 was isolated from a vector containing a 11.9kb *Spe*I-*Apa*I genomic fragment (1). Subsequently, a stop cassette containing an adenoviral splice acceptor (SA), a loxP-flanked hygromycin-thymidine kinase cassette (loxP-HygroTK-loxP), a poly adenylation signal from the PGK gene with two poly adenylation signals from SV40 (triple polyA: tpA), and a Tet operator with minimal CMV promoter (Tet) were integrated into the *Hin*dIII site in the 9.2kb *Sma*I-*Apa*I fragment. The expression vector pPyCAG-EGFP-IZ (2) was gifted from Hitoshi Niwa (Kumamoto Univ., Japan) and provided by the RIKEN BRC through the National Bio-Resource Project of the MEXT, Japan. For generating the expression vector pPyCAG-H3.3wt-FH-IZ, EGFP cassette was removed from pPyCAG-EGFP-IZ, and replaced to a synthesized DNA H3.3-Flag-HA. For generating pPyCAG-K36M-FH-IZ, point mutation of lysine (K) 36 to methionine (M) (AAG to ATG) were integrated into pPyCAG-H3.3wt-FH-IZ vector taking advantage of standard recombinant PCR method.

Chromatin immunoprecipitaiton (ChIP) for H2AK119ub1

ChIP for H2AK119ub1 was performed as previously described (3) with some modifications. Anti-H2AK119ub1 antibody (Upstate, #05-678) and normal mouse IgM (Sigma M5909) for a mock control were used as ChIP antibodies. Each genotype of ES cells was trypsinized, counted and 2 x 10^6 of each cells were resuspended in 1ml of ES medium. Formaldehyde was added to a final concentration of 1%, and the samples were incubated at room temperature for 5 min on a rotating wheel. The reaction was stopped by addition of glycine to a final concentration of 125 mM. After 5 min incubation on a rotating wheel, the cells were washed in ice-cold PBS twice before "swelling buffer" (25mM HEPES pH7.5, 1.5mM MgCl2, 10mM KCl and 0.1% NP-40, 1mM DTT, with complete, the protease inhibitor cocktail (#11 697 498 001, Roche, Mannheim, Germany) was added to lyse the cells. After 10min incubation at 4ºC, the nuclei were isolated by Dounce homogenization (20 strokes, "tight" pestle) and centrifuged. After resuspension in 100µl of "0.5% SDS sonication buffer" (50mM Hepes pH7.5, 140mM NaCl, 1mM EDTA, 1% Triton X-100, 0.1% Na-deoxycholate, 0.5% SDS) for 20min at 4ºC, the DNA was sonicated using a Covaris S1 system with following settings: Duty 20%; Intensity 10.0; Cycles/burst 500; Duration sec. 360; 1 cycle. The resulting material was centrifuged, and the supernatant was transferred to new tubes with 900ul of "0.05% SDS sonication buffer" (50mM

Hepes pH7.5, 140mM NaCl, 1mM EDTA, 1% Triton X-100, 0.1% Na-deoxycholate, 0.05% SDS with complete) for adjusting the final concentration of SDS as around 0.1%. 3μg of each H2AK119ub1 antibody and normal mouse IgM were added to each of 450μl of sonicated chromatin, and incubated at 4ºC overnight on a rotating wheel. 45μl of chromatin (1/10 volume) were used as an input fraction. Chromatin precipitated with anti-H2AK119ub1 or mock antibodies were purified by incubating for another 3 hours at 4ºC with anti-mouse IgM antibody raised in goat (M8644, Sigma), which had been previously bound to Dynabead-Protein-G (100.04D, Invitrogen, California, USA). The chromatin-bound beads were then washed twice in "sonication buffer" (50mM Hepes pH7.5, 140mM NaCl, 1mM EDTA, 1% Triton X-100, 0.1% Na-deoxycholate, 0.1%SDS), once in "washing buffer" (2mM Tris pH8.0, 0.02mM EDTA, 50mM LiCl, 0.1% NP-40, 0.1% Na-deoxychorate) and once in TE buffer. Elution of immunoprecipitated chromatin and extraction of DNA were carried out as previously described (4).

Knockdown experiment of the *Setd2* gene

J1 ES cells were cultured as previously described (5). Knockdown experiment was performed

using siSetd2 (Mouse Setd2, #L-062392-00, ON-TARGET plus SMART pool, Thermo Scientific, Massachusetts, USA) or siCtrl (ON-TARGET plus Control pool, #D-001810-10-05, Thremo Scientific) with lipofection reagent RNAiMAX following the manufacturer's protocol (#13778075, Thermo Scientific). In brief, 0.3 millions of J1 cells were put onto laminin-coated 35mm dishes containing siRNA-lipid complexes, composed of 100pmol of siRNA and 5μl of RNAiMax in Opti-MEM. Forty-eight hours after transfection, the cells were detached and transfected with the siRNA again using the same condition of the first transfection. Forty-eight hours after the transfection (totally 96 hours after the first transfection), the cells were collected for the downstream experiments including ChIP, qRT-PCR and RNA-FISH.

Supplemental References

1. **Sado T, Wang Z, Sasaki H, Li E.** 2001. Regulation of imprinted X-chromosome inactivation in mice by Tsix. Development **128:**1275-1286.
2. **Niwa H, Toyooka Y, Shimosato D, Strumpf D, Takahashi K, Yagi R, Rossant J.** 2005. Interaction between Oct3/4 and Cdx2 determines trophectoderm differentiation. Cell **123:**917-929.
3. **Stock JK, Giadrossi S, Casanova M, Brookes E, Vidal M, Koseki H, Brockdorff N, Fisher AG, Pombo A.** 2007. Ring1-mediated ubiquitination of H2A restrains poised RNA polymerase II at bivalent genes in mouse ES cells. Nat Cell Biol **9:**1428-1435.
4. **Sado T, Hoki Y, Sasaki H.** 2005. Tsix silences Xist through modification of chromatin structure. Dev Cell **9:**159-165.
5. **Ying QL, Wray J, Nichols J, Batlle-Morera L, Doble B, Woodgett J, Cohen P, Smith A.** 2008. The ground state of embryonic stem cell self-renewal. Nature **453:**519-523.
6. **Leeb M, Wutz A.** 2007. Ring1B is crucial for the regulation of developmental control genes and PRC1 proteins but not X inactivation in embryonic cells. J Cell Biol **178:**219-229.
7. **Wutz A, Jaenisch R.** 2000. A shift from reversible to irreversible X inactivation is triggered during ES cell differentiation. Mol Cell **5:**695-705.
8. **Shibata S, Yokota T, Wutz A.** 2008. Synergy of Eed and Tsix in the repression of Xist gene and X-chromosome inactivation. EMBO J **27:**1816-1826.
9. **Mikkelsen TS, Ku M, Jaffe DB, Issac B, Lieberman E, Giannoukos G, Alvarez P, Brockman W, Kim TK, Koche RP, Lee W, Mendenhall E, O'Donovan A, Presser A, Russ C, Xie X, Meissner A, Wernig M, Jaenisch R, Nusbaum C, Lander ES, Bernstein BE.** 2007. Genome-wide maps of chromatin state in pluripotent and lineage-committed cells. Nature **448:**553-560.
10. **Huynh KD, Lee JT.** 2003. Inheritance of a pre-inactivated paternal X chromosome in early mouse embryos. Nature **426:**857-862.
11. **Chureau C, Chantalat S, Romito A, Galvani A, Duret L, Avner P, Rougeulle C.** 2011. Ftx is a non-coding RNA which affects Xist expression and chromatin structure within the X-inactivation center region. Hum Mol Genet **20:**705-718.

12. **Tian D, Sun S, Lee JT.** 2010. The long noncoding RNA, Jpx, is a molecular switch for X chromosome inactivation. Cell **143:**390-403.

13. **Ohhata T, Senner CE, Hemberger M, Wutz A.** 2011. Lineage-specific function of the noncoding Tsix RNA for Xist repression and Xi reactivation in mice. Genes Dev **25:**1702-1715.

14. **Ohhata T, Hoki Y, Sasaki H, Sado T.** 2008. Crucial role of antisense transcription across the Xist promoter in Tsix-mediated Xist chromatin modification. Development **135:**227-235.

15. **Leeb M, Pasini D, Novatchkova M, Jaritz M, Helin K, Wutz A.** 2010. Polycomb complexes act redundantly to repress genomic repeats and genes. Genes Dev **24:**265-276.

16. **Nimura K, Ura K, Shiratori H, Ikawa M, Okabe M, Schwartz RJ, Kaneda Y.** 2009. A histone H3 lysine 36 trimethyltransferase links Nkx2-5 to Wolf-Hirschhorn syndrome. Nature **460:**287-291.

17. **Navarro P, Chambers I, Karwacki-Neisius V, Chureau C, Morey C, Rougeulle C, Avner P.** 2008. Molecular coupling of Xist regulation and pluripotency. Science **321:**1693-1695.

Supplemental figure legends

Figure S1 Generation and characterization of Δ*Tsix* male ES cells in the Ring1b$^{-/-}$ background (R$^{-/-}$Δ*Tsix*). (**A**) *Tsix* genomic locus was targeted in the clone36 Ring1b$^{-/-}$ ES cells (6) and its parental line, J1:rtTA ES cells containing a tetracycline inducible transactivator (7). Clone36 Ring1b$^{-/-}$ cells have an inducible *Xist* cDNA transgene (Tg) under control of a tetracycline-inducible promoter on chromosome 11. This *Xist* Tg has been shown not to be expressed unless induced with doxycycline (6, 8). The genomic structure of the TST allele (Δ*Tsix*) is shown below the overall structure of *Xist*/*Tsix* loci. SA: splicing acceptor, pAs: triple polyA cassette, Tet: Tet operator with CMV minimal promoter. (**B**) Targeting scheme for generating the TST allele. A: *Apa*I. H: *Hin*dIII. K: *Kpn*I. S: *Sma*I. (**C**) Homologous recombination in Ring1b$^{-/-}$ cells was confirmed by Southern blotting (R$^{-/-}$TST^{2lox}). Excision of the hygromycin resistance cassette was also confirmed in the same blot (R$^{-/-}$TST). (**D**) Map of the *Xist*/*Tsix* locus with the transcriptional termination sites of X$^{\Delta}$Y and TST. The region used for quantitative real-time PCR (*Tsix* 3’ end, Fig. S1E) is also shown. (**E**) Quantitative real-time PCR analysis for 3' end of *Tsix* expression (left panel). R$^{-/-}$TST1 and 2 are independent clones. In X$^{\Delta}$Y Eed$^{-/-}$, TST and R$^{-/-}$TST cells, antisense transcription in the *Xist* promoter region was efficiently reduced to under 0.5%. Means and standard deviations are presented from three

independent experiments. Albeit, the site of transcriptional termination of *Tsix* differs between the $X^{\Delta}Y$ Eed$^{-/-}$ (8) and TST allele (Fig. S1D), *Tsix* function was disrupted in both cases. To simplify, the previous names are substituted as indicated in the right box. (**F**) Immunofluorescence analysis of Ring1b in undifferentiated ES cells with indicated genotypes. Over 95% of cells show clear nuclear signal of Ring1b protein in J1:rtTA, Δ*Tsix* and E$^{-/-}$Δ*Tsix* cells. None of the cells show Ring1b expression in Ring1b$^{-/-}$ and R$^{-/-}$Δ*Tsix* cells (n=50). (**G**) Western blot analysis of histone modifications by PRC1 (H2AK119ub1) and PRC2 (H3K27me3) in cells of the indicated genotypes. In Ring1b$^{-/-}$ and R$^{-/-}$Δ*Tsix* cells, H2AK119ub1 was highly reduced because of the loss of Ring1b protein. In E$^{-/-}$Δ*Tsix* cells, H3K27me3 was highly reduced because of the loss of the Eed protein. H3 was used as a loading control.

Figure S2 H3K36me3 is installed along with *Tsix* transcrption. A map of *Xist*/*Tsix* transcription unit (upper panel) and the result of ChIP-seq using H3K36me3 antibody in undifferentiated ESCs cultured in serum LIF medium from USSC genome browser (NCBI36/mm8) (lower panel) are shown (9).

Figure S3 The expression of H3.3wt and H3.3K36M is confirmed by anti H3 antibody. One microgram of acid-extracted histones (double the amount of Fig. 7B) were used for anti-H3 western blotting. Long exposure enabled to observe Flag-tagged H3.3 histones (open triangle) in H3.3wt and H3.3K36M-expressing cells.

Figure S4 No *Xist* accumulation is observed by RNA-FISH in H3.3K36M-expressing cells. *Xist* and *Tsix* RNA-FISH were performed in J1 cells expressing EGFP (control), H3.3wt (wildtype) and K36M. The percentage of *Tsix* or *Xist* positive nuclei revealed by RNA-FISH is shown (n>100).

Figure S5 *Xist* is derepressed in Setd2 knockdown cells. Setd2 or control siRNA were transfected into J1 ES cells. In the Setd2 knockdown cells, Setd2 mRNA expression confirmed by qRT-PCR (**A**) as well as H3K36me3 modification at the *Xist* promoter and exon1 confirmed by ChIP (**B**) were decreased to around 70% of its control (Setd2 mRNA: 68%, **:$p<0.01$, n=3, H3K36me3 at the *Xist* promoter: 70%, *Xist* exon1: 68%). The Setd2 knockdown did not significantly affect *Tsix* transcription confirmed by RNA-FISH (**C**) (n>100) and qRT-PCR (**D**)

(p=0.14, n=3). *Xist* acculumation was not observed by *Xist* RNA-FISH (n>100) in Setd2 knockdown cells (**C**). However, *Xist* expression was significantly increased in Setd2 knockdown cells (**D**) (2.15 times, *: $p<0.05$, n=3). Expression is shown relative to J1 cells transfected with control siRNA and normalized to Gapdh (**A** and **D**) (n=3). Sox2 gene body and its promoter for H3K36me3 were used as positive and negative control loci, respectively (**B**). All cells were cultured with 2i medium.

Table S1. PCR primer sequences for quantitative PCR

PCR products	Primer sequence	Intron	Expected length	Application	References
**Rnf12**		Yes	379bp	qRT-PCR (Fig. 1C)	(10)
Rlim-F	GAGCCCCGATGAAAATAGAGC				
Rlim-R	GGTCGGCACTTCTGTTACTGC				
**Xpct**		No	153bp	qRT-PCR (Fig. 1C)	
Xpct-1(+)20	ATTCGCCTTTCAACCATCAC				
Xpct-1(-)20	TGGGCCAGCTTGATTTTATC				
**Cnbp2**		No	128bp	qRT-PCR (Fig. 1C)	
Cnbp2-1(+)20	ATTTGGGCACATCCAGAAAG				
Cnbp2-1(-)20	GATGTCCAGACTCTCCACAG				
Ftx		Yes	84bp	qRT-PCR (Fig. 1C)	(11)
FtxE8.9Up	CTTGATTCAGCAACACATGAGGA				
FtxE10.9Lo	TCCAGGCAAGAGGGACCAG				
Jpx		No	131bp	qRT-PCR (Fig. 1C)	(12)
e1-F	GCACCACCAGGCTTCTGTAAC				
e1-R	GGGCATGTTCATTAATTGGCCAG				
Xist		Yes	173bp	qRT-PCR (Fig. 1C, 4C, 5C, 7E, S5D)	(13)
Xist-6(+)20	TCATCACAACAGCAGTTCTC				
Xist-8(-)20	CAGGAGCACAAAACAGACTC				
Tsix		No	122bp	qRT-PCR (Fig. 1C, 5C, 7E, S1E, S5D)	(14)
Xist (-540)F	ATGGAGTCACCAGGTTCCCA				
Xist-10 (-)20	ACAAAATGGCTCCTTGGTTC				
**Gapdh**		No	370bp	qRT-PCR (Fig. 1C, 4C, 5C, 7E, S1E, S5A, S5D)	(4)
Gapd F	ATGGCCTTCCGTGTTCCTAC				
Gapd R2	ATAGGGCCTCTCTTGCTCAG				
***Hoxa7* promoter**		No	151bp	ChIP-qPCR (Fig. 2B, 4D, 4E, 5B, 7D)	(3)
HOXA7 Promoter F	GAGAGGTGGGCAAAGAGTGG				
HOXA7 Promoter R	CCGACAACCTCATACCTATTCCTG				

***Oct4* promoter**		No	88bp	ChIP-qPCR (Fig. 2B, 4D, 4E, 5B, 7D)	(3)
Oct4 promoter F	GGCTCTCCAGAGGATGGCTGAG				
Oct4 promoter R	TCGGATGCCCCATCGCA				
***Gapdh* promoter**		No	176bp	ChIP-qPCR (Fig. 2C)	(15)
Gapdh promoter FW	AGCATCCCTAGACCCGTACAGT				
Gapdh promoter RV	GGGTTCCTATAAATACGGACTGC				
***H1 foo* promoter**		No	123bp	ChIP-qPCR (Fig. 2C)	(16)
H1foo-promoter-s	AGGCTAGCAGTAGTCTGGATCAG				
H1foo-promoter-as	ACTGTGTCCTACCTACCTGACGAG				
***Rnf12* promoter**		No	78bp	ChIP-qPCR (Fig. 2B, 2C)	
Rnf12P-1(+)20	GGCTCAACTGGGATTGGCTG				
Rnf12P-1(-)20	CACAACAGGAAGTGATGCTG				
***Xpct* promoter**		No	79bp	ChIP-qPCR (Fig. 2B, 2C)	
XpctP-1(+)20	CAGAGCTTGAGACCTGGGAC				
XpctP-1(-)20	CACTCAAACCAGACATTTAG				
***Ftx* promoter**		No	71bp	ChIP-qPCR (Fig. 2B, 2C)	
FtxP-1(+)20	GGAGAGGCTGTGGGTTCGTG				
FtxP-1(-)21	GATGAACACCAGGTACGAAAC				
***Jpx* promoter**		No	83bp	ChIP-qPCR (Fig. 2B, 2C, 5B, 6B)	
JpxCTCF-F2	CGGTCACGTTGCTGGAGCAG				
JpxCTCF-R2	TGCGTCACAGCCGAACAGTC				
***Xist* promoter**		No	209bp	ChIP-qPCR (Fig. 2B, 2C, 4D, 4E, 5B, 6B, 6C, 7C, 7D, S5B)	
Xist (-281)F	AGGTCACACACCTGTCTATG				
Xist (-489)R	GAGCGTAAGCCCACCAAATC				
***Xist* ex1**		No	87bp	ChIP-qPCR (Fig. 2B, 2C, 3B, 3C, 5B, 6B, 7C, 7D, S5B)	(17)
XEx1aF	CCACCCAACCAACCCAAT				
XEx1aR	TGGGCTTGGGATAGGTCTGA				
***Xist* int1**		No	79bp	ChIP-qPCR (Fig. 2B, 2C, 3B, 3C, 5B, 6B)	(17)
XIn1 F	AACCCTTTTAAGTCCACTGTAAATTCC				
XIn1 R	TAGAGAGCCAGACAATGCTAAGCC				

***Klf2* promoter**		No	100bp	ChIP-qPCR (Fig. 3B)	Unpublished data
U-Klf2	CTGCACAAAGGGCTTAGAGG				
D-Klf2	CCTCATTTGCACCACACCTA				
***Nanog* promoter**		No	130bp	ChIP-qPCR (Fig. 3C)	Unpublished data
U-Nanog	GCAGCCGTGGTTAAAAGATG				
D-Nanog	GAAGCTGTAAGGTGACCCAGA				
***Sox2* gene body**		No	127bp	ChIP-qPCR (Fig. 6B, 6C, 7C, S5B)	
SOX2-GB(+)20	CAGTACAACTCCATGACCAG				
SOX2-GB(-)20	CCTCGGACTTGACCACAGAG				
***Sox2* promoter**		No	92bp	ChIP-qPCR (Fig. 6B, 6C, 7C, S5B)	(3)
Sox2 promoter F	CCATCCACCCTTATGTATCCAAG				
Sox2 promoter R	CGAAGGAAGTGGGTAAACAGCAC				
Setd2		Yes	169bp	qRT-PCR (Fig. S5A)	
Setd2-2(+)20	GATCTGGATGGCAGAGCTTG				
Setd2-2(-)20	CTGAGGAACAGCAGTCTTAG				

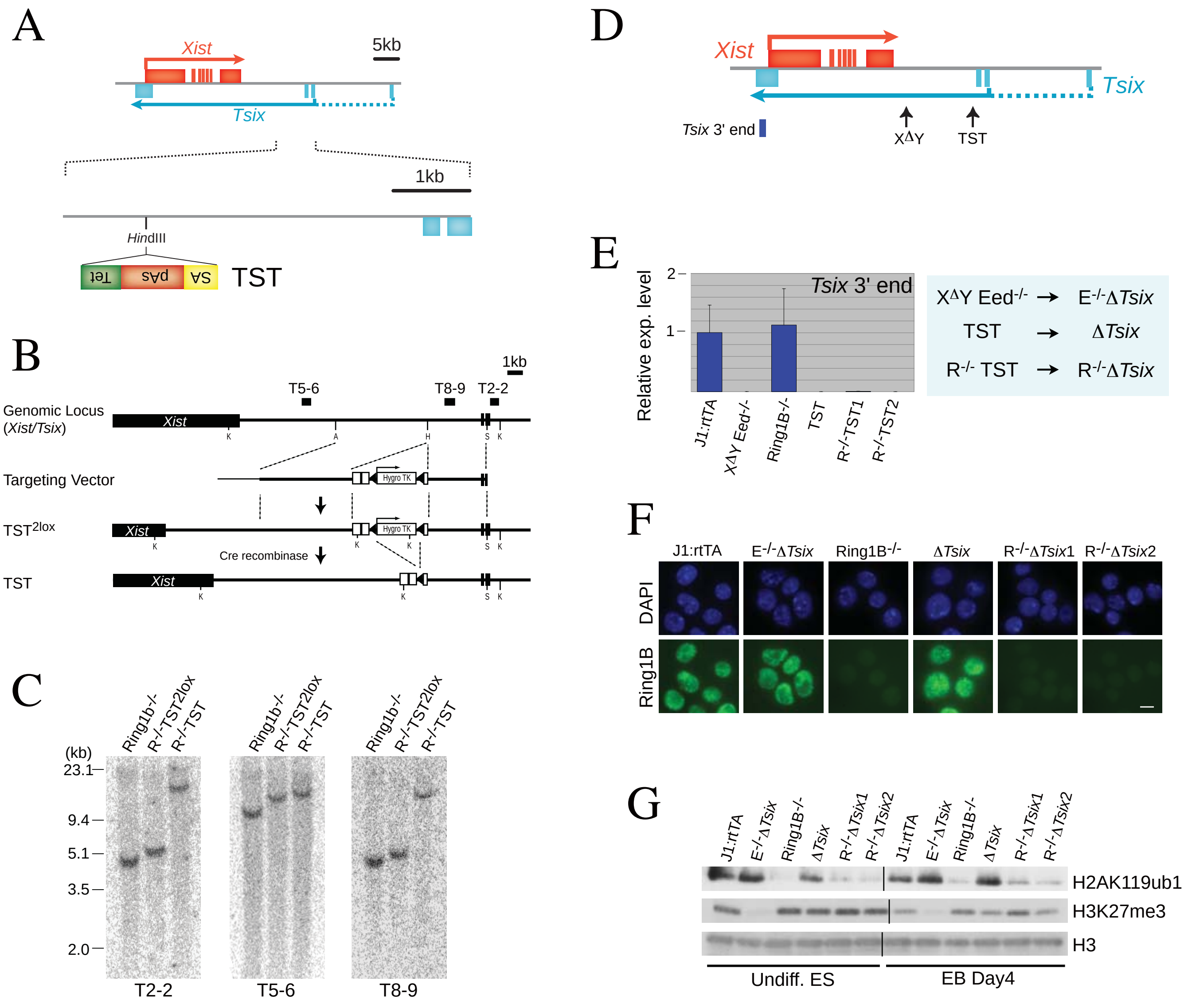

Ohhata T. et al., Fig S1

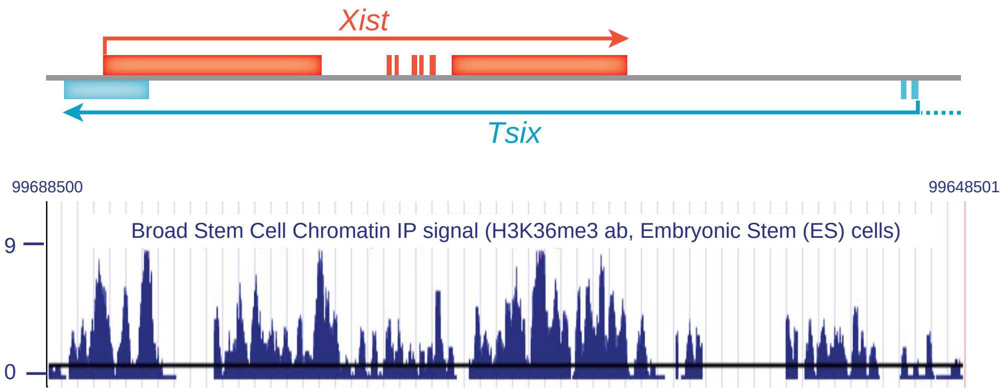

USSC Genome Browser on Mouse Feb. 2006 (NCBI36/mm8) Assembly

Mikkelsen TS et al., Nature, 2007.

Ohhata T. et al., Fig S2

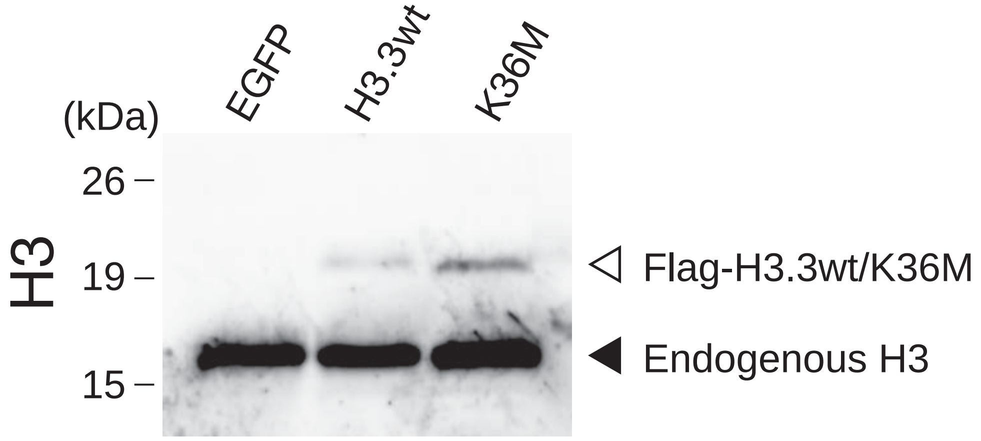

Ohhata T. et al., Fig S3

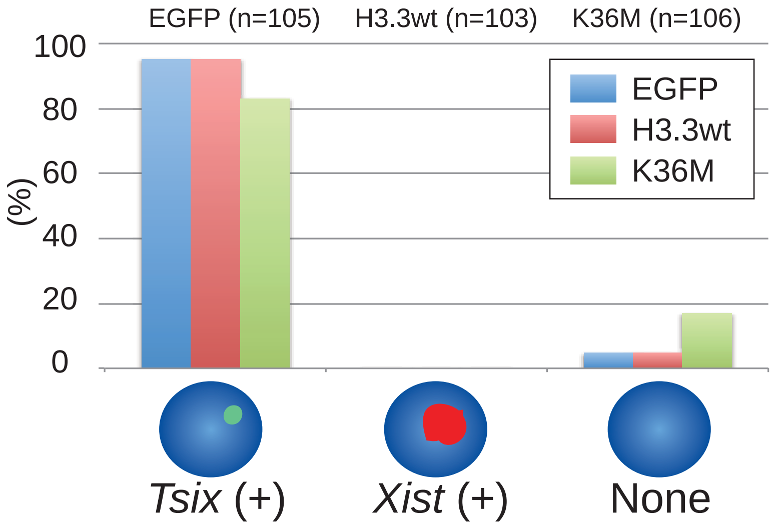

Ohhata T. et al., Fig S4

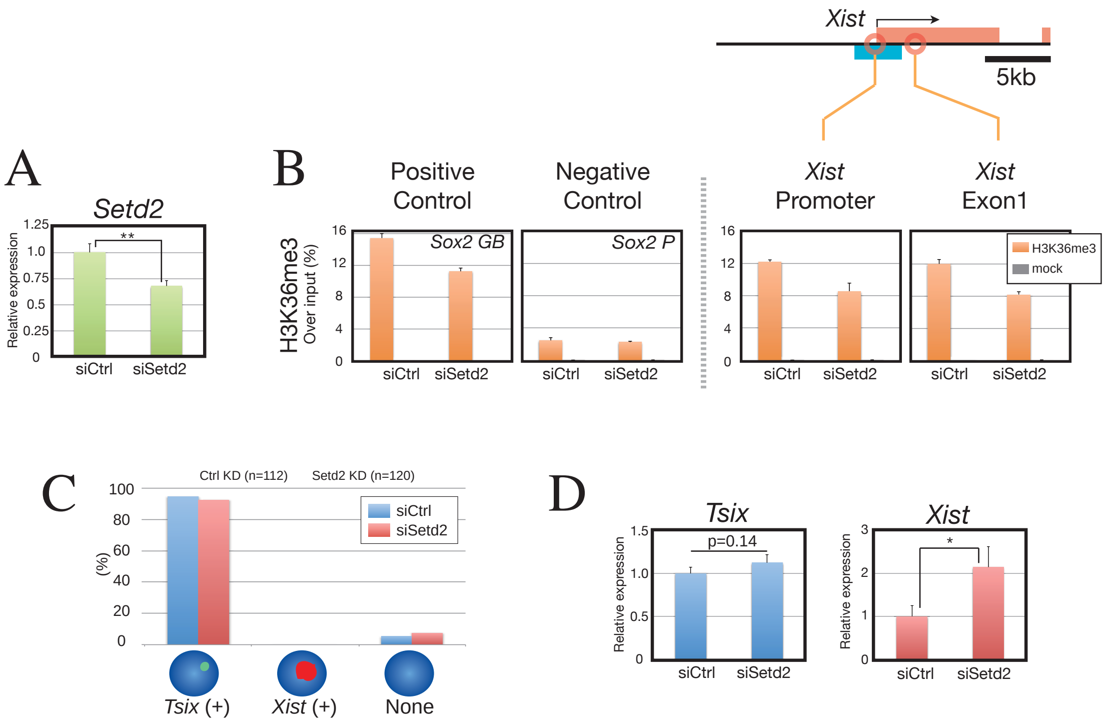

Ohhata T. et al., Fig S5
